# Hypoxic preconditioning increases mitochondrial respiration and H_2_O_2_ production

**DOI:** 10.3389/fnmol.2025.1628567

**Published:** 2025-11-19

**Authors:** Lisa Bergmeister, Carolina Doerrier, Barbara Fogli, Tímea Komlódi, Amrei Fischer, Kerstin Springer, Christoph Schwarzer, Erich Gnaiger

**Affiliations:** 1 Department of Pharmacology, Medical University of Innsbruck, Innsbruck, Austria; 2 Oroboros Instruments, Innsbruck, Austria

**Keywords:** mitochondria, high-resolution respirometry, reactive oxygen species, hypoxia, hypoxic preconditioning, epilepsy

## Abstract

**Introduction:**

Hypoxia, an inadequate tissue oxygen supply, poses a threat to the brain, which relies heavily on oxygen for its energy requirements. However, mild oxygen deficiency triggers cellular stress, leading to a defensive state known as hypoxic preconditioning (HPC). Despite its potential as a treatment option for neurodegenerative diseases, research on preconditioning remains a challenge. Therefore, this study aimed to further explore biochemical changes induced by HPC, with a specific emphasis on mitochondria, the primary oxygen consumers.

**Methods:**

We assessed the neuroprotective impact of a HPC protocol used by examining the seizure thresholds of mice. Additionally, we analyzed mitochondrial respiration under varying oxygen levels, reactive oxygen species (ROS) production, and mitochondrial morphology following HPC treatment.

**Results:**

HPC treatment of mice raised their seizure threshold, indicating an enhanced resistance to epileptic seizures and highlighting the protective effects of the HPC protocol. HPC increased mitochondrial oxygen consumption and ROS production, primarily originating from Complex I. Importantly, ROS levels remaining within the physiological range potentially activate cell signaling pathways. Our findings underscored the importance of controlling oxygen at physiologically relevant intracellular tissue levels (intracellular tissue normoxia) during mitochondrial respiration measurements. Notably, HPC-treated mitochondria generally exhibited reduced oxygen consumption compared to controls under effectively hyperoxic air-saturated oxygen conditions. However, mitochondrial respiration was increased under intracellular tissue normoxia in comparison to the controls measured at air saturation. Moreover, following HPC treatment, we observed alterations in mRNA expression levels associated not just with mitochondrial dynamics but also with perinuclear mitochondrial accumulation and pro-survival signaling. Furthermore, an immediate increase in mitochondrial fusion was observed following hypoxia treatment.

## 1 Introduction

The brain utilizes approximately 20% of the body’s resting metabolic energy even though it makes up just 2% of the total body mass (Mink et al., 1981). Unlike muscle cells, neurons lack significant energy reserves (Peters, 2011), making them highly susceptible to cognitive function declines with even brief energy interruptions. Thus, precise regulation of brain energy metabolism is vital. Both glycolysis and oxidative phosphorylation (OXPHOS) supply the brain with the required ATP (Murali Mahadevan et al., 2021). OXPHOS in mitochondria generates ATP when sufficient oxygen (O_2_) is available, but during conditions of hypoxia, the brain switches to the less effective processes of glycolysis, resulting in reduced ATP levels (Matsuda et al., 1992; Kopp et al., 1984; Rogatzki et al., 2015). OXPHOS accounts for up to 98% of the body’s O_2_ consumption (Silver and Erecinska, 1998), emphasizing the dependence of mitochondria on a continuous O_2_ supply for energy transformation.

The O_2_ concentrations within the body can vary depending on the specific anatomical location and are typically lower than the levels found in the atmosphere (21%). Arterial blood typically contains O_2_ levels of about 14%, while the brain’s O_2_ concentration is approximately 4%, decreasing with depth. The term “intracellular tissue normoxia” describes the O_2_ pressure that is considered physiological, whereas “functional hypoxia” indicates O_2_ levels that effectively limit respiration. Conversely, “environmental normoxia” is defined as the O_2_ level equivalent to that found in the atmosphere (Donnelly et al., 2022).

Functional hypoxia results in cellular adataptions of energy metabolism, mitochondrial respiration, nutrient utilization, and protein synthesis. As energy supplies are reduced during hypoxia, cells must relocate their energy metabolism and reduce ATP-consuming processes in order to survive (Lee et al., 2020). Furthermore, DNA, RNA, and protein synthesis are among the first processes to be inhibited during hypoxia (Buttgereit and Brand, 1995). However, the translation of transcripts that are essential for survival are enhanced, allowing cells to acclimate to low O_2_ conditions (van den Beucken et al., 2011).

During functional hypoxia, electron flow through the electron transfer system (ETS) slows down, discussed controversially as increasing (Thomas and Ashcroft, 2019) or decreasing (Komlodi et al., 2021) the risk of generating reactive oxygen species (ROS). Low levels of ROS contribute to the regulation of growth factor signaling, hypoxic responses, immune functions, and autophagy, however excessive ROS can cause oxidative harm (Reczek and Chandel, 2015; Azzi, 2022; Sies, 2020; Brand, 2016). Elevated ROS exposure can damage lipids, proteins, and DNA, resulting in cellular and mitochondrial dysfunction and reduced cell viability (Redza-Dutordoir and Averill-Bates, 2016; Thomas and Ashcroft, 2019; Sies, 2015). The brain is particularly susceptible to oxidative stress due to its rich mitochondrial content (especially in neurons), high O_2_ demands, limited damage repair capacity, and abundant polyunsaturated fatty acids (Pearson-Smith and Patel, 2017). Oxidative stress is closely linked to neurodegenerative diseases, e.g., Parkinson’s and Alzheimer’s, as well as acute brain injuries stemming from prolonged seizures, strokes, and traumatic incidents (Pearson-Smith and Patel, 2017; Zhang et al., 2017; Bhatti et al., 2018; Puspita et al., 2017; Cheignon et al., 2018). Mitochondria are an important source of endogenous ROS production, with the ETS being the site where ROS generation predominantly occurs (Tirichen et al., 2021; Brand, 2010). The superoxide anion (O_2_^•-^) is the most common ROS generated in mitochondria and is primarily produced at Complex I (CI) and Complex III (CIII) sites (Tahara et al., 2009; Tirichen et al., 2021). CI-derived O_2_^•-^ enters the mitochondrial matrix and rapidly converts to hydrogen peroxide (H_2_O_2_), which can then be released into the cytosol. On the other hand, O_2_^•-^ produced by CIII is primarily released into the intermembrane space, where it can either be converted into H_2_O_2_ and then released into the cytosol, or it can traverse the mitochondrial outer membrane and transform into H_2_O_2_ within the cytosol (Hoehne et al., 2022; Bleier et al., 2015; Muller et al., 2004). O_2_^•-^ originating from CI can also be produced via a process called reverse electron transfer (RET), which takes place when electrons return from ubiquinol to CI, reducing NAD^+^ to NADH (Scialo et al., 2017; Komlódi et al., 2018a,b). While both O_2_^•-^ and H_2_O_2_ serve as signaling molecules, H_2_O_2_ is recognized as the primary ROS signaling molecule, and it can selectively oxidize target molecules at physiological concentrations (Sinenko et al., 2021; Sies and Jones, 2020).

O_2_ levels have an impact on mitochondrial morphology, with mitochondria appearing tubular during tissue normoxia and fragmented during hypoxia (Lee et al., 2020). The morphology of mitochondria varies according to their function and cellular requirements, and different physiological and pathophysiological conditions affect their shape (Tilokani et al., 2018). Mitochondrial fission is vital for distributing mitochondria during cell division, apoptosis, intracellular transfer, and eliminating damaged mitochondria. In contrast, mitochondrial fusion enables the exchange of essential components, creating a more uniform network capable of rescuing compromised mitochondria (Yapa et al., 2021; Westermann, 2010). The process of mitochondrial fission is orchestrated by the GTPase dynamin-related protein 1 (DRP1). In contrast, mitochondrial fusion involves GTPases such as mitofusin 1 and 2 (MFN1/2) for fusion of the mitochondrial outer membrane and optic atrophy 1 (OPA1) for mitochondrial inner membrane fusion (Macdonald et al., 2016; Akepati et al., 2008). Interestingly, models of neurodegenerative diseases, e.g., Alzheimer’s, Parkinson’s, and epilepsy show changes in mitochondrial structure. For instance, Alzheimer’s and Parkinson’s disease models display increased mitochondrial fragmentation, while kainic acid epilepsy models exhibit mitochondrial swelling (Wang et al., 2008; Kudin et al., 2002; Yang et al., 2021). Furthermore, owing to their distinctive cellular morphology, neurons require mitochondria to travel extended distances. Hypoxia can prompt the redistribution of mitochondria within cells, leading to their accumulation around the nucleus. This mitochondrial relocation is observed in both short- and long-term hypoxia and is believed to play a role in delivering mitochondrial ROS to the nucleus. These ROS trigger oxidative modifications in the promoter regions of genes under hypoxia-inducible factor 1 (HIF1) regulation, subsequently amplifying gene expression (Al-Mehdi et al., 2012).

Diminished O_2_ levels are detected through multiple pathways, with HIFs playing a vital role in maintaining O_2_ balance by selectively controlling the expression of numerous genes in a tissue-specific manner (Liu et al., 2021; Semenza, 1999). HIFs govern a range of functions such as cell survival, proliferation, apoptosis, metabolism, energy production, and pro- and antioxidative responses (Ostrowski and Zhang, 2020). Additionally, factors like mitochondrial ROS can activate the HIF pathway, even under normal O_2_ conditions (Chandel et al., 2000; Sanjuan-Pla et al., 2005), a phenomenon referred to as pseudohypoxia (Hou et al., 2014).

Hypoxia contributes to various neurological diseases, e.g., epilepsy, Parkinson’s disease, and Alzheimer’s disease and is a major cause of neurological disabilities and strokes (Burtscher et al., 2021; Shukitt-Hale et al., 1998; Silva et al., 2016). Nevertheless, when exposed to mild oxygen deficiency, cells can undergo stress, triggering a response that offers protection or builds tolerance – a phenomenon referred to as hypoxic preconditioning (HPC). The concept of HPC is that a mild and transient reduction in O_2_ levels can prompt cellular defenses and enhance the ability to acclimate to varying O_2_ conditions (Dahl and Balfour, 1964; Stetler et al., 2014). Many *in vitro* and *in vivo* studies demonstrate the neuroprotective attributes of HPC, with several factors suggested to contribute to the advantageous outcomes of HPC (Silva et al., 2016; Zhan et al., 2010; Thompson et al., 2013; Pan et al., 2014; Yang et al., 2013; Zhang et al., 2006; Turovskaya et al., 2020; Zhen et al., 2014). Preconditioning can manifest within two forms: rapid or delayed HPC. The former one occurs within minutes to hours, involving alterations in enzyme activity, protein phosphorylation, post-translational modifications, secondary messengers, ion channels, and expression of immediate early genes, whereas the latter one takes hours or days, ultimately leading to alterations in gene expression and the synthesis of new proteins (Zhang et al., 2006; Turovskaya et al., 2020; Li et al., 2017; Barone et al., 1998; Kirino, 2002).

Epilepsy is a prevalent neurological condition known for its persistent susceptibility to spontaneous seizures, causing significant physical, psychological, social, and economic challenges for individuals and society (Fisher et al., 2014). Although anti-seizure drugs (ASDs) are the primary epilepsy treatment, nearly 30% of patients cannot achieve seizure control (Chen et al., 2018). Even those who respond to ASDs frequently still suffer from occasional seizures, encounter therapy side effects, and experience a gradual decline in functional abilities (Thijs et al., 2019). As a result, researchers have delved into alternative therapies with neuroprotective potential to enhance clinical outcomes (Silva et al., 2016; Zhen et al., 2014; Curia et al., 2014). Numerous studies have demonstrated the neuroprotective benefits of HPC, including its capacity to mitigate seizure susceptibility, frequency, and severity, as well as its role in diminishing neuronal loss and enhancing cognitive function (Gao et al., 2014; Silva et al., 2016; Turovskaya et al., 2020; Yang et al., 2013; Pohle and Rauca, 1994; Gao et al., 2012; Rauca et al., 2000; Emerson et al., 1999; Rubaj et al., 2000; Zhang et al., 2006).

The treatment options for epilepsy are not the only ones facing limitations. But the treatment of other neurodegenerative diseases, e.g., Alzheimer’s and Parkinson’s disease encounters constraints, underscoring the pressing need for the development of additional treatment options (Silva et al., 2016; Thijs et al., 2019; Pereira, 2016; Long et al., 2021). Despite its potential as a treatment approach for neurodegenerative diseases, the field of preconditioning still has remained understudied. Therefore, it is essential to delve deeper into the mechanisms that take place following HPC, as they may be accountable for adataption and tolerance, especially important in the brain, where neurons cannot regenerate (Rybnikova and Samoilov, 2015; Stetler et al., 2014). This study aimed to investigate and provide further insights into the biochemical changes induced by HPC, with a specific emphasis on mitochondria, the hub of O_2_ consumption.

Initially, we evaluated the neuroprotective capacity of the HPC protocol employed by assessing the seizure threshold in an acute seizure mouse model. Next, we explored the impact of varying O_2_ levels on mitochondrial respiration in brain tissue samples from both untreated and HPC-treated mice using high-resolution respirometry. Additionally, we examined alterations in mitochondrial respiration, ROS production and dynamics following mild hypoxia treatment in brain tissue samples. For this purpose, we created a Macro compatible with Fiji-ImageJ software, simplifying the analysis of mitochondrial morphology.

The results outlined in this study have already been made accessible online through LB’s Ph.D. thesis (Bergmeister, 2023).

## 2 Materials and methods

### 2.1 Animals

We used wild-type C57BL6/J male mice (aged 10–25 weeks). Mice were split into two groups: control (Ctrl) and HPC treated. They were housed under standard conditions (12/12 light/dark cycles, 23 °C), with up to five mice per ventilated type II L cage, providing food and water *ad libitum*. Cages were enriched with bedding, nesting materials, and a mouse house. This study was approved by the Animal Ethics Committee of the Medical University Innsbruck and the Austrian Animal Experimentation Ethics Board (in accordance with EU Directive 2010/63/EU). We ensured that the number of mice utilized for the experiments was minimized to the greatest extent possible.

### 2.2 Hypoxic preconditioning

**Figure 1:**
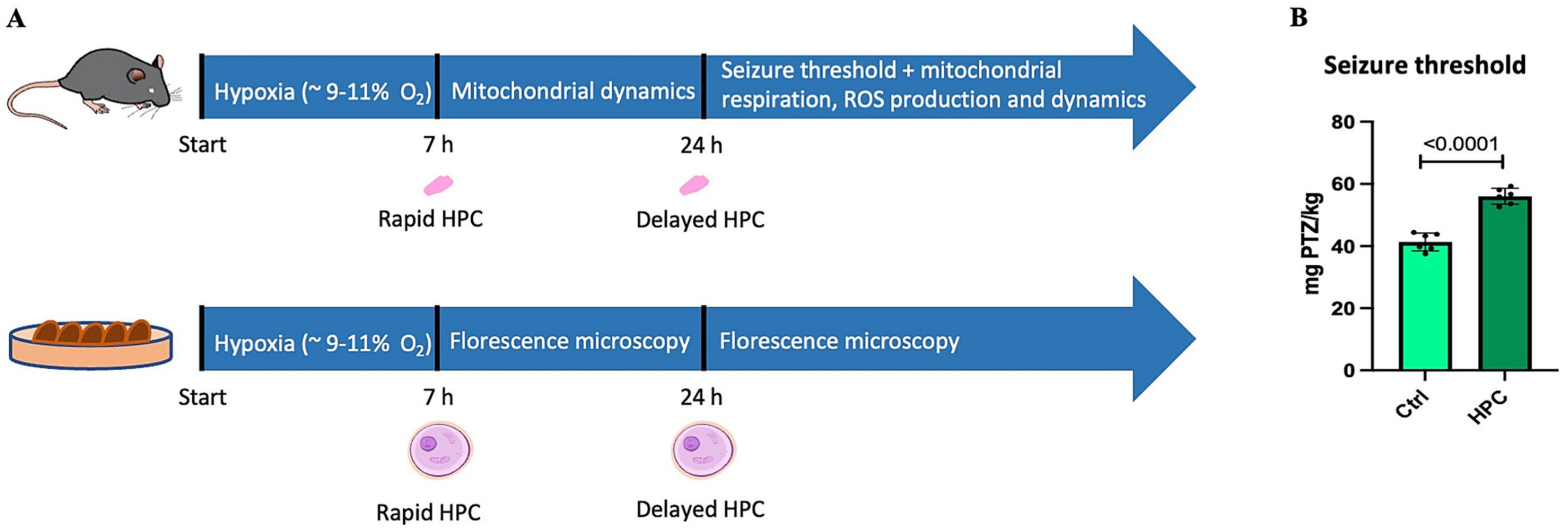
Experimental workflow and the influence of hypoxic preconditioning (HPC) on the seizure threshold. **(A)** In the *in vivo* part of the present study, C57BL6/J mice underwent 7 h of hypoxia exposure (HPC). Immediately after hypoxia (rapid HPC), brain tissue samples were collected for qPCR analysis to assess the expression of key factors related to mitochondrial fusion and fission. Twenty-four hours after initiation of the hypoxic phase (17 h after hypoxia, delayed HPC), the seizure threshold was measured and qPCR analyses were conducted to examine the expression of the same genes associated with mitochondrial fusion and fission. Moreover, mitochondrial respiration and ROS production were analyzed. Mitochondrial respiration was assessed under both air-saturated O_2_ levels and conditions of intracellular tissue normoxia. For the *in vitro* part, CHO cells were subjected to 7 h of hypoxia treatment. Immediately after hypoxia and 17 h later, mitochondria were stained, the cells were fixed, and fluorescence microscopy analysis to evaluate mitochondrial morphology was performed. The image of the mouse featured in the upper section of the figure (https://doi.org/10.5281/zenodo.6645614) and the single cell displayed in the lower part of the figure (https://doi.org/10.5281/zenodo.3926537) were both obtained from SciDraw. **(B)** The seizure thresholds in naive and HPC-treated mice were determined by administering the GABA_A_ receptor antagonist PTZ (pentylenetetrazole) until a seizure occurred. The seizure threshold was defined as the quantity of PTZ infused (mg) per kg of the mouse’s body weight. The findings are expressed as the mean ± SD. Statistical analysis was performed using a t-test, with the significance level set at ≤ 0.05.

Mice were subjected to a gas mixture consisting of 9% O_2_ and 91% N_2_ (Linde Gas) for 7 h. For hypoxia treatment, up to 5 mice were placed in a new, airtight cage. These cages are equipped with air inlet and outlet valves, one valve was utilized to deliver the gas mixture, and the other one to monitor O_2_ levels using an O_2_ meter (Apogee Instruments). The measured O_2_ concentration in the cages reached levels between 9 and 12% O_2_. The flow meter (Fyearfly) was set to maintain a flow rate of 2–3 L/min, refreshing air 15–20 times per hour. Mice were monitored hourly during the 7-h hypoxia. Figure 1A illustrates the workflow of the present study. Immediately after hypoxia exposure (referred to as rapid HPC), samples were gathered for subsequent qPCR analyses (section 3.7) to evaluate the expression of genes linked to mitochondrial dynamics. Additionally, a further set of assessments was carried out 24 h after the initiation of hypoxia (referred to as delayed HPC). These assessments encompassed the determination of seizure thresholds (section 3.3), measurement of mitochondrial respiration (section 3.5.1 and 3.5.2), evaluation of H_2_O_2_ generation (section 3.5.2), and gene expression analyses (qPCR) targeting the same genes associated with mitochondrial dynamics.

### 2.3 Seizure threshold

To test the neuroprotective potential of HPC we evaluated the seizure thresholds in both naive and HPC-treated mice (*N* = 6/group), by applying the GABA_A_ receptor antagonist pentylenetetrazole (PTZ). Following the measurement of body mass, mice were restrained for cannula insertion into the tail vein. Continual infusion of PTZ (10 mg PTZ/mL 0.9% NaCl, pH 7.4) was performed in freely moving mice until inducing a tonic–clonic seizure. The seizure threshold was determined based on the PTZ volume infused per kg of mouse. Infusion occurred at 100 μL/min, with a maximum of 250 μL administered. After mice experienced a generalized seizure, they were killed with a neck dislocation.

### 2.4 Brain tissue preparation

Tissue preparation followed the protocol by Burtscher et al. (2015) with some adjustments. Naive and HPC-treated mice (*N* = 6–15 mice) were killed through cervical dislocation. Brains were swiftly harvested, with dissections focusing on either the hippocampus, motor cortex, and striatum, or only the hippocampus and striatum. Samples from the hippocampus, motor cortex, and striatum were utilized for measurements of mitochondrial respiration under air-saturated O_2_ levels (section 3.5.1) and gene expression analyses (section 3.7). However, due to constrained availability of O2k-FluoRespirometers (Oroboros Instruments), concurrent measurements of mitochondrial respiration and H_2_O_2_ production under brain tissue normoxia (section 3.5.2) were carried out exclusively with the hippocampus and striatum. Brain tissues were immediately placed in an ice-cold mitochondrial respiration medium (MiR05Cr, 20 mM creatine (Cr), without bovine serum albumin/BSA, Oroboros Instruments). After washing three times in MiR05Cr without BSA, tissues were quickly dried on blotting paper (GE Healthcare Life Science), weighed (Mettler Toledo AE 160), and approximately 6 mg of wet tissue mass were suspended in 500 μL of respiration medium. The remaining tissue was frozen (−80 °C) for qPCR analyses. Homogenization was conducted using a pre-cooled glass homogenizer (DWK Wheaton and tight fit; WiseStir homogenizer HS-30E, witeg Labortechnik GmbH) at 1000 rpm with 10 strokes for the motor cortex and striatum, and 15 strokes for the hippocampus. We measured mitochondrial respiration and H_2_O_2_ production of homogenates from 2 mg of the wet tissue mass, suspended in mitochondrial respiration medium in air-calibrated 2 mL Oroboros chambers (calibrated with MiR05Cr with BSA). Each measurement had two technical replicates. Post-measurement, samples were collected from each chamber and frozen (−80 °C) for later citrate synthase (CS) activity determination.

### 2.5 High-resolution respirometry (HRR)

#### 2.5.1 HRR at air-saturated O_2_ concentrations

**Figure 2:**
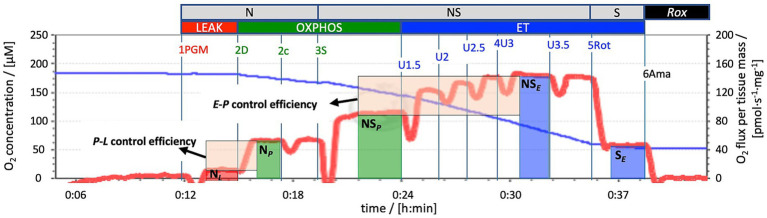
Mitochondrial respiration using a SUIT protocol (SUIT-028). Representative trace of mitochondrial respiration using a Substrate-Uncoupler-Inhibitor Titration (SUIT) protocol. The blue line represents O_2_ concentration [μM], the red line O_2_ flux per tissue wet mass [pmol_·_s^−1^_·_mg^−1^]. The assessment of mitochondrial respiration was conducted using high-resolution respirometry (Oroboros FluoRespirometer), with data recorded using DatLab 7 software. Measurement was performed at O_2_ levels near air saturation. 1PGM: Initial addition of pyruvate (P; 5 mM), malate (M, 2 mM), and glutamate (G; 10 mM) to induce NADH- (N)-linked LEAK respiration N*_L_*. 2D: A kinetically saturating ADP concentration (D; 2.5 mM) induced N-linked OXPHOS N*_P_*. 2*c*: Cytochrome *c* (c; 10 μM) added to assess the integrity of the mitochondrial outer membrane**. 3S**: CII-substrate succinate (S; 50 mM) added to evaluate NS-pathway OXPHOS capacity NS*_P_* (CI- and CII-linked). 4U: Stepwise titrations of the uncoupler carbonyl cyanide chlorophenylhydrazone CCCP (U; 1.5–3.5 μM) to reach maximum respiration as an estimate of NS-pathway ET capacity NS*_E_*. 5Rot: Inhibition of CI by rotenone (Rot; 0.5 μM) to measure S-linked ET capacity S*_E_*. 6Ama: Inhibition of CIII by antimycin A (Ama; 2.5 μM) to assess residual oxygen consumption *Rox* for baseline correction of O_2_ flux in all mitochondrial respiratory states. The vertical bars for N*_L_* (red), N*_P_* and NS*_P_* (green), NS*_E_* and S*_E_* (blue) indicate the sections marked for calculation of respiratory flux. The shaded boxes provide a graphical explanation of the ratios (arrows): *P*-*L* control efficiency = (N*_P_*-N*_L_*)/N*_P_* = 1-N*_L_*/N*_P_*; *E*-*P* control efficiency = (NS*_E_*-NS*_P_*)/NS*_E_* = 1-NS*_P_*/NS*_E_*.

Mitochondrial function was evaluated using Oroboros FluoRespirometers (Oroboros Instruments, Innsbruck, Austria) and applying a Substrate-Uncoupler-Inhibitor Titration (SUIT) protocol. O_2_ concentration [μM] and O_2_ flux per tissue mass [pmol O_2·_s^-1·^mg^−1^] were measured real-time using DatLab 7.4 software at 37 °C near air-saturated O_2_ conditions (150–200 μM, reoxygenations were performed by opening the Oroboros chambers). Samples from the hippocampus, motor cortex, and striatum were analyzed. The SUIT protocol utilized (Figure 2) followed the methodology outlined by Burtscher et al. (2015) and Burtscher et al. (2018) involving multiple steps to assess different respiratory states. The NADH (N)-linked LEAK state (N*_L_*) was initiated by introducing pyruvate (P; 5 mM), malate (M, 2 mM), and glutamate (G; 10 mM). Activating the phosphorylation system, with kinetically saturating ADP concentrations (D; 2.5 mM), enabled the assessment of OXPHOS through the N-linked pathway (N*_P_*). In the OXPHOS state, the CII-linked substrate succinate (S; 50 mM) was employed to assess the convergent electron flow into the coenzyme Q through the NS pathway (NS*_P_*). The electron transfer (ET) capacity for the NS-linked pathway (NS*_E_*) was determined by CCCP (carbonyl cyanide m-chlorophenyl hydrazone) uncoupler titration (U; 1.5–3.5 μM). Careful titration of uncouplers was undertaken to identify optimal concentrations for maximal O_2_ flux assessment. Further, the CI inhibitor rotenone (Rot; 0.5 μM) was introduced to measure S-linked ET capacity (S*_E_*), and finally the CIII inhibitor antimycin A (Ama; 2.5 μM) was used to quantify residual O_2_ consumption (*Rox*). O_2_ fluxes were corrected for *Rox* and normalized for wet tissue mass to determine the mass-specific O_2_ flux. The activity of citrate synthase (CS [IU]) was used as a mitochondrial marker to express respiration as mitochondrial (mt)-specific O_2_ flux (Burtscher et al., 2015; Doerrier et al., 2018; Gnaiger, 2020; Gnaiger et al., 2020).

Flux control ratios (*FCR*s) were calculated by normalizing O_2_ consumption in each state for the maximum O_2_ flux (NS*_E_*), allowing for evaluation of mitochondrial respiratory control patterns irrespective of mitochondrial content. Additional control ratios were calculated (Figure 2B): *E-P* control efficiency and *P-L* control efficiency. *E-P* control efficiency (1-NS*_P_*/NS*_E_*) evaluates the limitation of OXPHOS capacity by the phosphorylation system. A value of 0 implies that OXPHOS capacity is not limited by the phosphorylation system (equal OXPHOS and ET capacities, *P* = *E*), while values above 0 indicate an increasing control over OXPHOS capacity by the phosphorylation system (OXPHOS capacity being lower than ET capacity, *P* < *E*). *P*-*L* control efficiency (1-N*_L_*/N*_P_*) of 1 indicates a fully coupled system, while 0 indicates no coupling (Gnaiger, 2020; Burtscher et al., 2018; Burtscher et al., 2015).

#### 2.5.2 HRR under physiologically relevant O_2_ levels

Oroboros FluoRespirometer in combination with the green fluorescence sensors were used to study mitochondrial respiration and H_2_O_2_ generation at physiologically relevant O_2_ levels (30–40 μM, reoxygenations were performed by opening the Oroboros chambers). Homogenates from the hippocampus and striatum were analyzed. The SUIT protocol outlined in section 3.5.1 was implemented. O_2_ concentration [μM], and O_2_ and H_2_O_2_ fluxes per tissue mass [pmol O_2·_s^-1·^mg^−1^] and [pmol H_2_O_2·_s^-1·^mg^−1^], respectively, were measured real-time using DatLab 7.4 software at 37 °C. The Amplex® UltraRed (AmR) assay was used to measure the net production of H_2_O_2_, with emitted fluorescence correlating directly to the amount of H_2_O_2_ converted to the fluorescent product UltroxRed (Komlodi et al., 2021). The amperometric channel voltage was set to 500 mV and the gain adjusted to 1,000. First, background H_2_O_2_ calibration was carried out in the absence of biological sample to determine the chemical background fluorescence and AmR sensitivity. This calibration was performed by titrating H_2_O_2_ in the presence of the chelator diethylenetriamine-*N, N, N′, N, N*-pentaacetic acid (DTPA), superoxide dismutase (SOD), horseradish peroxidase (HRP), and AmR. Afterward, samples were added into the chambers. During the experiment, H_2_O_2_ titration steps (0.1 μM) were performed to monitor changes in fluorescence sensitivity over time. The H_2_O_2_ calibration solution was prepared fresh every day (Komlódi et al., 2018a,b). At the beginning of the experiment, either H_2_ or N_2_ was introduced into the gas phase of the open chamber to lower the O_2_ concentration, allowing the experiment to be performed at reduced O_2_ levels. The traces presented in Supplementary Figures S1A,B show exemplary measurements of mitochondrial respiration and H_2_O_2_ production.

O_2_ fluxes were corrected and normalized as detailed in section 3.5.1. H_2_O_2_ fluxes were corrected for background fluorescence and AmR sensitivity (Komlodi et al., 2021). H_2_O_2_ fluxes were normalized to the wet tissue mass and CS activity to determine mass-specific and mitochondria-specific H_2_O_2_ flux. Additionally, the *J*_H2O2_/*J*_O2_ ratio was calculated, representing H_2_O_2_ produced per O_2_ consumed.

Initially, tests were conducted to assess the influence of AmR and fluorescence light on mitochondrial respiration by comparing measurements in the presence/absence of AmR and fluorescence light. As changes were noted in mitochondrial respiration after hypoxia treatment due to AmR and fluorescence light presence (Supplementary Figures S2–S4), separate measurements were taken for H_2_O_2_ and O_2_ fluxes from the same samples.

### 2.6 Citrate synthase (CS) activity

CS catalyzes the transformation of acetyl-CoA and oxaloacetate into citrate and CoA-SH:

Acetyl-CoA + oxaloacetate + H_2_O = > citrate + CoA-SH.

The introduction of 5,5′-dithiobis-2-nitrobenzoic acid (DTNB) initiates the irreversible formation of thionitrobenzoic acid (TNB, absorption at 412 nm):

CoA-SH + DTNB = > TNB + CoA-S-S-TNB.

CS activity was determined by mixing the following components in a glass cuvette: 0.31 mM acetyl-CoA, 0.25% Triton X-100, 0.1 mM DTNB, 50 μL sample containing 2 mg/mL wet tissue mass, and distilled deionized water. The reaction was initiated by adding 0.5 mM oxalacetate. A glass cuvette containing distilled deionized water was used as blank. Acetyl-CoA was diluted in distilled deionized water, oxalacetate in 0.1 M triethanolamine-HCl-buffer with 1 mM EDTA (pH 8), and DTNB in Tris–HCl-buffer (pH 8.1). The latter two solutions were freshly prepared daily. Using a spectrophotometer (Hitachi), the linear absorption increase at 412 nm was measured every 10 s for 120 s. The change in absorption over time directly correlates with CS activity. Specific enzyme activity (*v*) was computed using the equation,


$$v=\frac{\mathrm{rA}}{l*\varepsilon_{B}*v_{B}}*\frac{V_{\text{cuvette}}}{V_{\text{sample}}*\rho }$$

*v* = specific activity per mg protein in international units (IU [μmol of citrate per min]).

*r_A_* = change of absorbance over time [min^−1^].

*l* = optical path length, 1 cm.

*ε*_B_ = extinction coefficient of TNB (B) at 412 nm and pH 8.1, 13.6 mM^−1^·cm^−1^.

*v*_B_ = stochiometric number of B (1).

*V*_cuvette_ = cuvette volume, 1 mL.

*V*_sample_ = sample volume added, 50 μL.

*ρ* = sample mass concentration [mg_·_mL^−1^] (Gnaiger, 1993).

### 2.7 Gene expression analysis

Tissue samples from the hippocampus, motor cortex, and striatum underwent gene expression analysis. Dynabeads® mRNA DIRECT™ Kit (Dynal) was used for mRNA extraction following the manufacturer’s protocol. Purified mRNA (150 ng) was utilized for first-strand cDNA synthesis with Jump StartTM REDTaq® ReadyMixTM Reaction Mix (Sigma). To determine the expression of target genes associated with mitochondrial dynamics (listed in Table 1) real-time qPCR analyses were conducted using 1 μL of generated cDNA plus specific primers (250 nM of forward and reverse primers). As a master mix the 5x HOT FIREPol EvaGreen qPCR Mix Plus (Solis Biodyne) was used. Measurements were conducted using a Bio-Rad CFX Connect Real-Time System Thermal Cycler, and results were analyzed with Bio-Rad CFX Manager. The ΔΔCt method (2^-ΔΔCt^) was applied to normalize the target gene expression to the reference gene (*Actb*), enabling a comparison of gene expression levels across different groups.

**Table 1:** Target genes and primer sequences.

Gene	Marker for	Primer sequence
*mMfn1*	Mitochondrial fusion	m*Mfn1* fwd CCGATGGAGATAAAGCCTACC
		m*Mfn1* rev CAAGAGGGCACATTTTGCTT
*mMfn2*	Mitochondrial fusion	m*Mfn2* fwd TCCCTCTCAAGCACTTTGT
		m*Mfn2* rev CCAGTTCTGTGTTCCTGTGG
*mDrp1*	Mitochondrial fission	m*Drp1* fwd CCAAAGTACCTGTAGGCGAT
		m*Drp1* rev CAGCAGTGACGGCGAGGATA
*mOpa1*	Mitochondrial fusion	m*Opa1* fwd CTATAAGTGGATTGTGCCTGA
		m*Opa1* rev GCAATCATTTCCAGCACACTG
*mActb*	Housekeeping gene	m*Actb* fwd CATTGCTGACAGGATGCAGAAGG
		m*Actb* rev TGCTGGAAGGTGGACAGTGAGG

All primers were purchased from Microsynth; fwd, forward; rev, reverse.

### 2.8 Staining of mitochondria in cell culture

Mitochondrial staining was conducted using CHO cells, comparing Ctrl cells to those exposed to 7 h of hypoxia. The stained cells were either fixed immediately after hypoxia treatment (rapid HPC) or 17 h later (delayed HPC). Cells were cultivated in DMEM/F-12 medium supplemented with fetal bovine serum (FBS; 10%), glutamax (1x), penicillin and streptomycin (100 U/mL), and G418 (400 μg/mL) at 37 °C and 5% CO_2_. Mitochondrial staining was conducted using 1 mM MitoTracker Red CMXRos dye dissolved in DMSO.

The day prior to the experiment, a total of 10^6^ cells were seeded in 10 cm^2^ dishes containing cover slips. To induce hypoxia, the cells were positioned in a hypoxia incubator chamber (Billups-Rothenberg Inc.), where the gas mixture (9% O_2_, 5% CO_2_, and 86% N_2_; Linde Gas) was introduced. Following this, the hypoxia chamber containing the cells was transferred into the incubator (Thermo Fisher Scientific) and maintained for a duration of 7 h. Hourly, measurements of the O_2_ concentration were made, and the gas mixture was refreshed.

To prepare for staining, both Ctrl and treated cells were rinsed with PBS, and then a colorless medium (DMEM medium without BSA and phenol red) was added. Subsequently, 1 μL of the 1 mM MitoTracker Red CMXRos stock solution was added and incubated for 45 min at 37 °C in the dark. Afterward, cells were washed twice with PBS and then fixed for 20 min at room temperature in the dark with a solution composed of paraformaldehyde (PFA, 3%) and glutaraldehyde (GA, 1.5%) dissolved in PBS at a pH of 7.4 (Qin et al., 2021). After fixation, cells were washed three additional times in PBS and the coverslips were mounted onto microscope slides by using Vectashield mounting medium.

**Figure 3:**
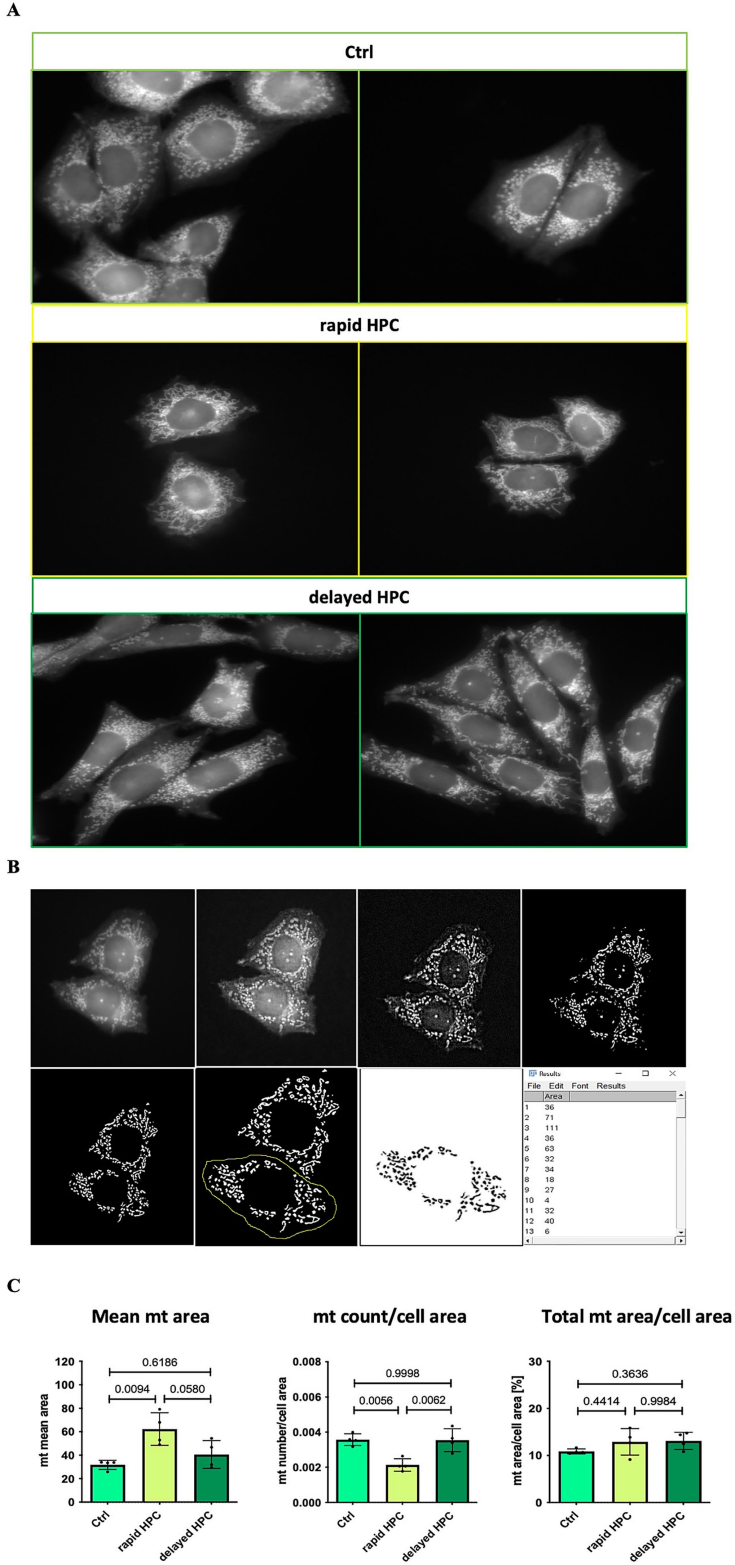
Effect of HPC-treatment on mitochondrial morphology *in vitro*. **(A)** Fluorescence microscopy images showcasing mitochondria stained with MitoTracker Red CMXRos in CHO cells, provided as representative examples. One million cells were cultured one day before the experiment. On the subsequent day, the Ctrl cells were left untreated, while the HPC cells underwent a 7-h hypoxia treatment. Afterward, mitochondria were stained, and cells were fixed. For the rapid HPC group, staining and fixation occurred immediately after hypoxia, while the delayed HPC group underwent these processes 17 h later. Imaging was conducted using a fluorescence microscope with a 63x resolution and immersion oil. **(B)** Figures illustrating the evaluation process of stained mitochondria in CHO cells. The evaluation was conducted utilizing Fiji-ImageJ, which included applying a Macro to improve contrast and define a threshold for detecting positive signals. False positives were manually removed. Following that, the cell of interest was encircled and examined to determine the quantity of particles (first column) and their corresponding areas (second column). Furthermore, the original image (first image) was employed to assess cell size. **(C)** The average mitochondrial area (size), mitochondrial number, and total mitochondrial area per cell were determined. To account for differences in cell sizes, the mitochondrial count and total mitochondrial area were adjusted relative to the cell’s size. The total mitochondrial area relative to cell area is expressed as a percentage. Data represented as means ± SD. We conducted a one-way ANOVA test with a significance threshold set at ≤ 0.05.

Images were captured using a fluorescence microscope (Zeiss; 63 x objective, 10 x magnification in camera tube) utilizing immersion oil. These images were subjected to analysis using Fiji-ImageJ software. In order to ensure unbiased assessment, samples were blinded for both microscopy analysis and subsequent evaluation. Four cover slips per condition were analyzed, from which three cells were chosen at random per cover slip. The average value derived from these three cells was employed for subsequent analysis. Mitochondria were automatically identified through a Macro we developed, as demonstrated in Figure 3B. This Macro improved contrast, established a threshold for positive signals (mitochondria), and eliminated background noise. However, the Macro had limitations, necessitating manual correction for false positives. Subsequently, the cell of interest was delineated, and particle number and size (area) were quantified. To further minimize the risk of false positives, particles equal to or smaller than an area of 3 (arbitrary unit) were excluded. Cell size, derived from the unmodified image, was used for normalization purposes. For each condition, the average mitochondrial size per cell, along with the quantity of mitochondrial count per cell area and mitochondrial area per cell area, was determined.

### 2.9 Summary of the data

In order to offer a comprehensive perspective on the influence of HPC throughout the entire brain, we aggregated the data collected from the assessed brain regions. The mean value from combined individual brain regions was utilized for conducting statistical analysis. Furthermore, specific data from individual brain regions can be found in the Supplementary materials.

### 2.10 Statistical analyses

Statistical analyses were performed using GraphPad Prism 9 software. Prior to evaluating statistical significance, the ROUT method was employed to identify outliers. Comparative analyses were conducted between two groups, Ctrl and HPC-treated mice, for seizure threshold, mitochondrial respiration, and H_2_O_2_ production. To evaluate the seizure threshold a *t-*test was utilized to compare means between the groups. A *p*-value of ≤ 0.05 was considered statistically significant, and results are presented as mean ± standard deviation (SD).

For both mitochondrial respiration and H_2_O_2_ production we employ nonparametric tests. The Mann–Whitney U test was used to compare group medians, with significance defined at ≤ 0.05. Results are expressed as median ± interquartile range (IQR).

The evaluation of mitochondrial dynamics encompassed three groups: Ctrl, rapid HPC, and delayed HPC. A one-way analysis of variance (ANOVA) test was administered to compare the means among the groups. Statistically significant results were considered with a *p*-value of ≤ 0.05, and findings are reported as mean ± SD. Additionally, the Šidák multiple comparisons test was applied to correct for multiple comparisons.

## 3 Results

### 3.1 Increased seizure threshold after HPC

To assess the neuroprotective capabilities of the HPC protocol, we examined seizure thresholds in both naive mice (N = 6) and those subjected to HPC treatment (*N* = 6) by applying the GABA_A_ receptor antagonist, PTZ. As depicted in Figure 1B, HPC treatment significantly elevated the seizure threshold in C57BL6/J mice.

### 3.2 Mitochondrial respiration and H_2_O_2_ production

#### 3.2.1 Mitochondrial respiration under conditions of air-saturated O_2_ concentrations

**Figure 4:**
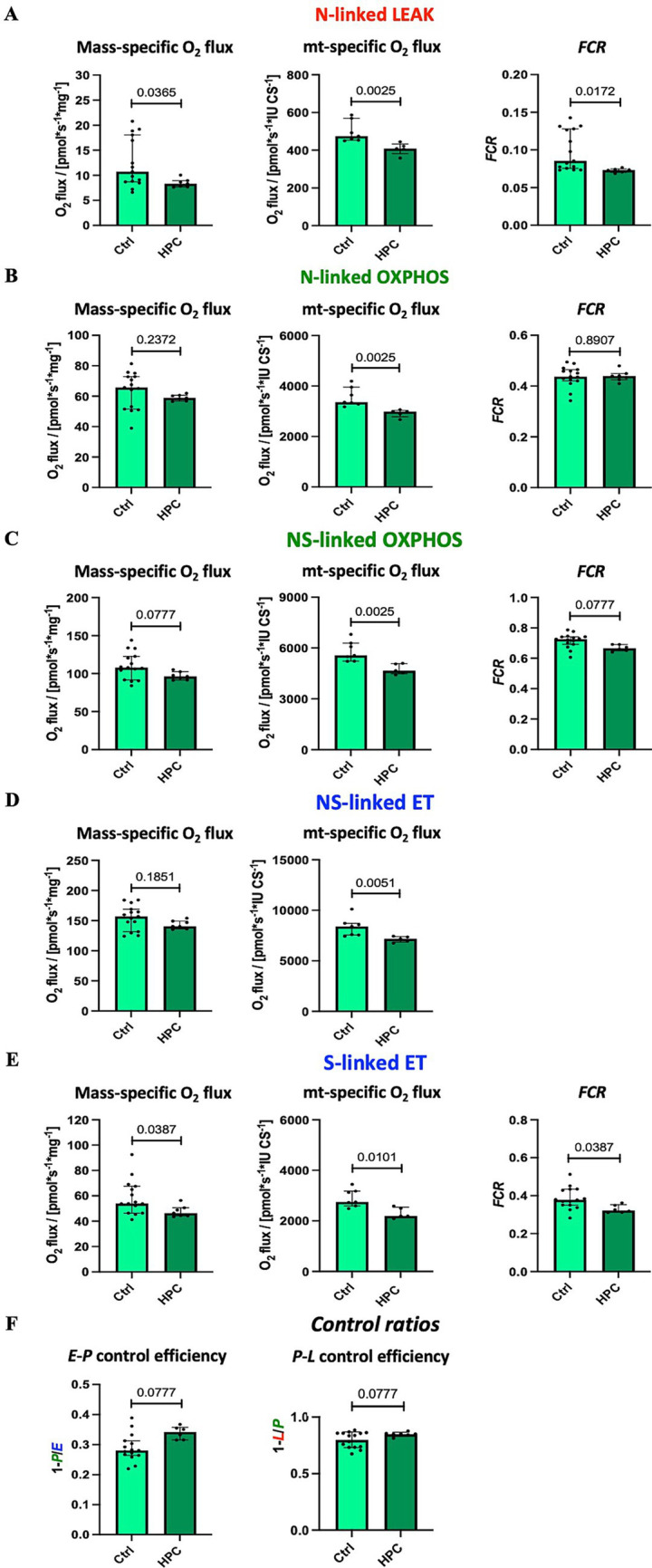
Effect of HPC-treatment on mitochondrial respiration measured near air-saturated O_2_ concentrations. Means of pooled data from hippocampus, motor cortex, and striatum. Mass-specific O_2_ flux [pmol_·_s^−1^_·_mg^−1^] was normalized for wet tissue mass. Citrates synthase activity (CS [IU]) was used as a mitochondrial marker for expression of mt-specific O_2_ flux [pmol_·_s^−1^_·_IU ^−1^]. Flux control ratios *FCR* were normalized for maximal O_2_ flux NS*_E_*. **(A)** NADH-linked LEAK respiration N*_L_*. **(B)** NADH-linked OXPHOS capacity N*_P_*. **(C)** NADH- & succinate-linked OXPHOS capacity NS*_P_*. **(D)** NADH- & succinate-linked ET capacity NS*_E_*. **(E)** Succinate-linked ET capacity S*_E_*. **(F)** Respirometric control efficiencies. See figure legend 2 for respiratory states and rates, and for definition of respirometric control efficiencies. Data represent medians ± IQR and statistical significance was determined using the Mann–Whitney U test, with the significance level set at ≤ 0.05. For mass-specific O_2_ flux and *FCR* 15 controls and 7 HPC treated mice were analysed. For CS-specific O_2_ flux 7 controls and 5 HPC treated mice were analysed. In the *E*-*P* control efficacy, the N-linked *FCR*, the NS-linked *FCR*, S-linked ET *FCR* 1 HPC treated mouse was identified as outlier and excluded from analysis.

Using high-resolution respirometry, we analyzed mitochondrial respiration across various respiratory states (Figure 2). In order to investigate the impact of different O_2_ levels on mitochondrial respiration, experiments were conducted near air saturation (150–200 μM O_2_) and O_2_ levels close to physiological intracellular tissue normoxia (30–40 μM) for brain tissue cells (Hadanny and Efrati, 2020). The pooled data, combining results for hippocampus, motor cortex, and striatum, on mitochondrial respiration near air-saturated O_2_ conditions are displayed in Figure 4. Supplementary Figures S5, S6 show the detailed unpooled data. For mass-specific O_2_ flux and *FCR* 15 controls (including controls from experiments with rapid HPC treated mice) and 7 delayed HPC treated mice were analysed. For citrate synthase specific O_2_ flux 7 controls and 5 HPC treated mice were analysed. In the *E*-*P* control efficacy, the N-linked *FCR*, the NS-linked *FCR*, S-linked ET *FCR* 1 HPC treated mouse was identified as outlier and excluded from analysis.

Citrate synthase activity was used as a matrix marker enzyme, and NS-linked ET capacity served as an internal functional marker to calculate flux control ratios. Under near air-saturated O_2_ levels post HPC treatment, N-linked LEAK respiration was significantly reduced in mass-specific and CS-specific O_2_ flux, as well as in the *FCR* (Figure 4A), when compared to the Ctrl group. Moreover, following HPC treatment, we noted a reduction in CS-specific O_2_ flux during N- and NS-linked OXPHOS (Figures 4B,C), alongside a decline in NS-linked ET capacity (Figure 4D). Nevertheless, there were no alterations in mass-specific O_2_ flux and in the *FCR* observed between the two groups (N- and NS-linked OXPHOS and NS-linked ET capacity). Furthermore, in comparison to Ctrl mice, HPC led to reductions in both mass-specific and CS-specific O_2_ flux, along with a diminished *FCR* of S-linked ET capacity (Figure 4E). *E-P* and *P-L* control efficiencies did not exhibit significant changes due to HPC (Figure 4F).

#### 3.2.2 Increased N-linked LEAK respiration after HPC under intracellular tissue normoxia

**Figure 5:**
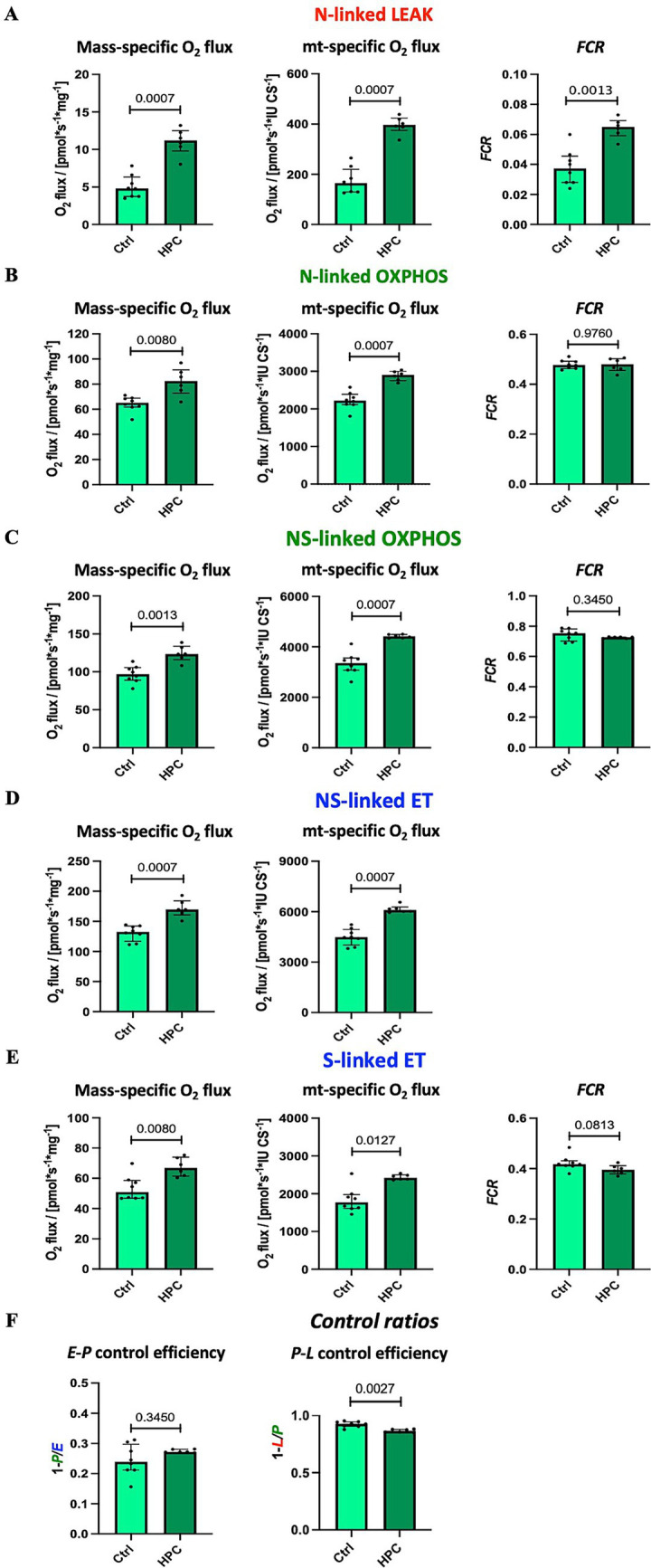
Effect of HPC-treatment on mitochondrial respiration assessed under tissue normoxia at O_2_ concentration of 30–40 μM. Means of pooled data for hippocampus and striatum. **(A)** NADH-linked LEAK respiration N*_L_*. **(B)** NADH-linked OXPHOS capacity N*_P_*. **(C)** NADH- & succinate-linked OXPHOS capacity NS*_P_*. **(D)** NADH- & succinate-linked ET capacity NS*_E_*. **(E)** Succinate-linked ET capacity S*_E_*. **(F)** Respirometric control efficiencies. See figure legend 2 for respiratory states and rates, and for definition of respirometric control efficiencies, and figure legend 3 for normalization of O_2_ flux. Data represent medians ± IQR and statistical significance was determined using the Mann–Whitney U test, with a threshold of ≤ 0.05 considered statistically significant. Eight control and 6 HPC treated mice were analysed throughout. For NS-linked *FCR* and *P-L* control efficiency one HPC treated mouse was identified and excluded from analysis.

The findings from the analysis of mitochondrial respiration performed under physiologically relevant O_2_ levels are illustrated in Figure 5. The results presented here represent pooled data, combining results for hippocampus and striatum. Eight control and 6 HPC treated mice were analysed throughout. For NS-linked *FCR* and *P-L* control efficiency one HPC treated mouse was identified and excluded from analysis. Unpooled data are available in Supplementary Figures S7, S8. In contrast to the outcomes observed near air-saturated O_2_ conditions, HPC induced significant elevations in mass-specific O_2_ fluxes, CS-specific O_2_ flux, and the *FCR* of N-linked LEAK when compared to the Ctrl (Figure 5A).

Furthermore, diverging from the results obtained near air-saturated O_2_ conditions, HPC treatment led to a significant increase in both mass-specific and CS-specific O_2_ flux during N- and NS-linked OXPHOS (Figures 5B,C), as well as N- and NS-linked ET capacity (Figures 5D,E) when compared to the Ctrl. The *FCR*s of these states remained unaffected by these changes.

The *E*-*P* control efficiency remained unaltered by HPC treatment. However, there was a significant decrease in *P*-*L* control efficiency within the HPC-treated group (Figure 5F).

#### 3.2.3 HPC treatment increased H_2_O_2_ production during N-linked LEAK and OXPHOS

**Figure 6:**
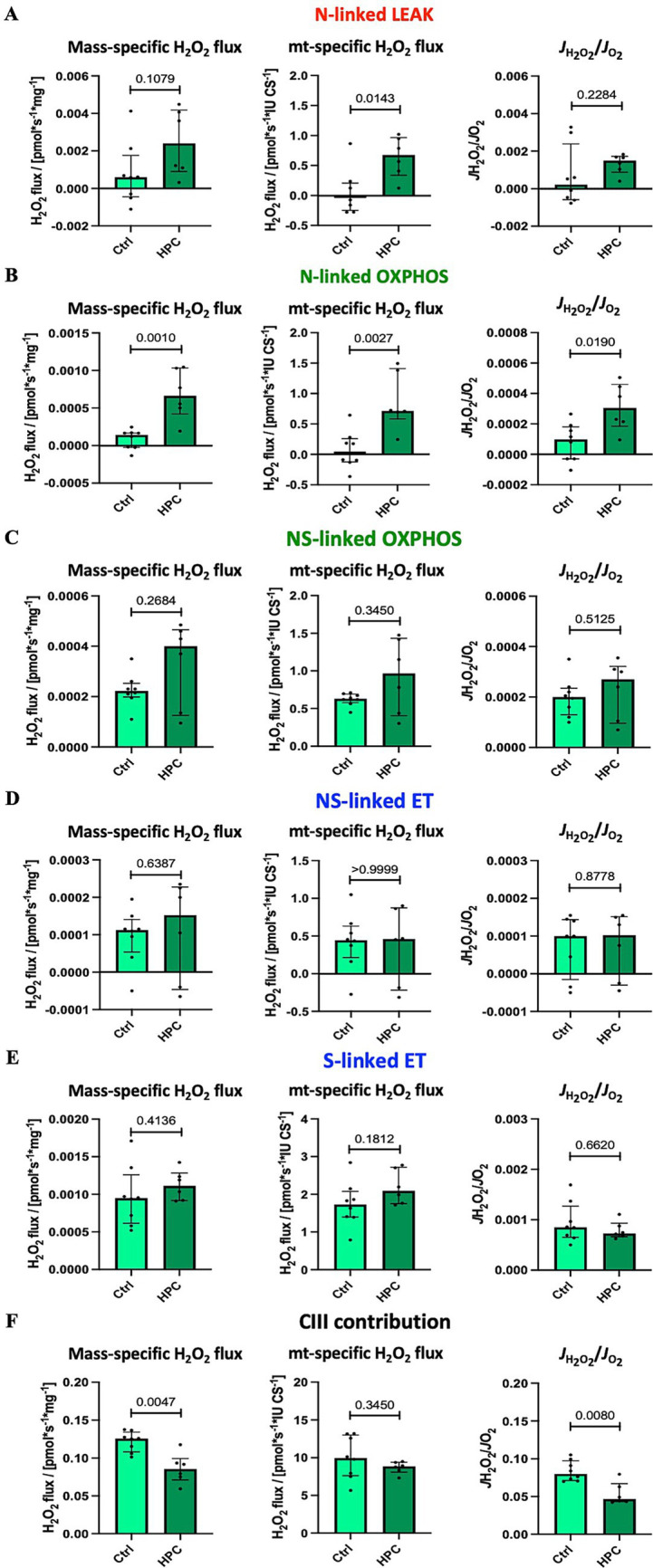
Effect of HPC-treatment on H_2_O_2_ production under tissue normoxia at O_2_ concentration of 30–40 μM. Means of pooled data for hippocampus and striatum. Net H_2_O_2_ flux measured in the following states: **(A)** NADH-linked LEAK N*_L_*. **(B)** NADH-linked OXPHOS N*_P_*. **(C)** NADH- & succinate-linked OXPHOS NS*_P_*. **(D)** NADH- & succinate-linked ET NS*_E_*. **(E)** Succinate-linked ET S*_E_*. **(F)** Maximal contribution of CIII to H_2_O_2_ production after adding the CIII inhibitor antimycin A. The *J*_H2O2_/*J*_O2_ ratio is the proportion of H_2_O_2_ produced to O_2_ consumed. H_2_O_2_ flux was normalized to O_2_ flux in the absence of AmR. See figure legend 2 for respiratory states and rates, and figure legend 3 for normalization of flux. Data represent medians ± IQR. Statistical test: Mann–Whitney U test, ≤ 0.05, was defined as statistically significant. Eight control and 6 HPC treated mice were analysed throughout. For N-linked mt-specific H_2_O_2_ flux one HPC treated mouse was identified and excluded from analysis.

H_2_O_2_ flux was measured under intracellular tissue normoxia (Supplementary Figure S1). The pooled results of ROS production measurements, combining results for hippocampus and striatum, are depicted in Figure 6 (unpooled data are available in Supplementary Figures S9, S10). 8 control and 6 HPC treated mice were analysed throughout. For N-linked mt-specific H_2_O_2_ flux one HPC treated mouse was identified and excluded from analysis. Following HPC, a significant increase in CS-specific H_2_O_2_ flux during LEAK respiration in the N-linked pathway was observed. However, HPC did not yield significant changes in LEAK respiration for either the mass-specific H_2_O_2_ flux or the ratio of H_2_O_2_ production to O_2_ consumption (*J*_H2O2_/*J*_O2_). Furthermore, significant elevations were observed after HPC in both mass-specific and CS-specific H_2_O_2_ fluxes, along with the *J*_H2O2_/*J*_O2_ ratio, during N-linked OXPHOS (Figure 6B). Conversely, H_2_O_2_ production during NS-linked OXPHOS (Figure 6C) and N- and NS-linked ET capacity (Figures 6D,E) displayed no significant changes. The application of Ama enabled the assessment of the maximum contribution of CIII to H_2_O_2_ production (Olowe et al., 2020). Mass-specific H_2_O_2_ production and *J*_H2O2_/*J*_O2_ significantly decreased following HPC, with no significant alteration in CS-specific H_2_O_2_ production.

### 3.3 Mitochondrial fusion and fission

#### 3.3.1 Changes in mRNA expression

**Figure 7:**
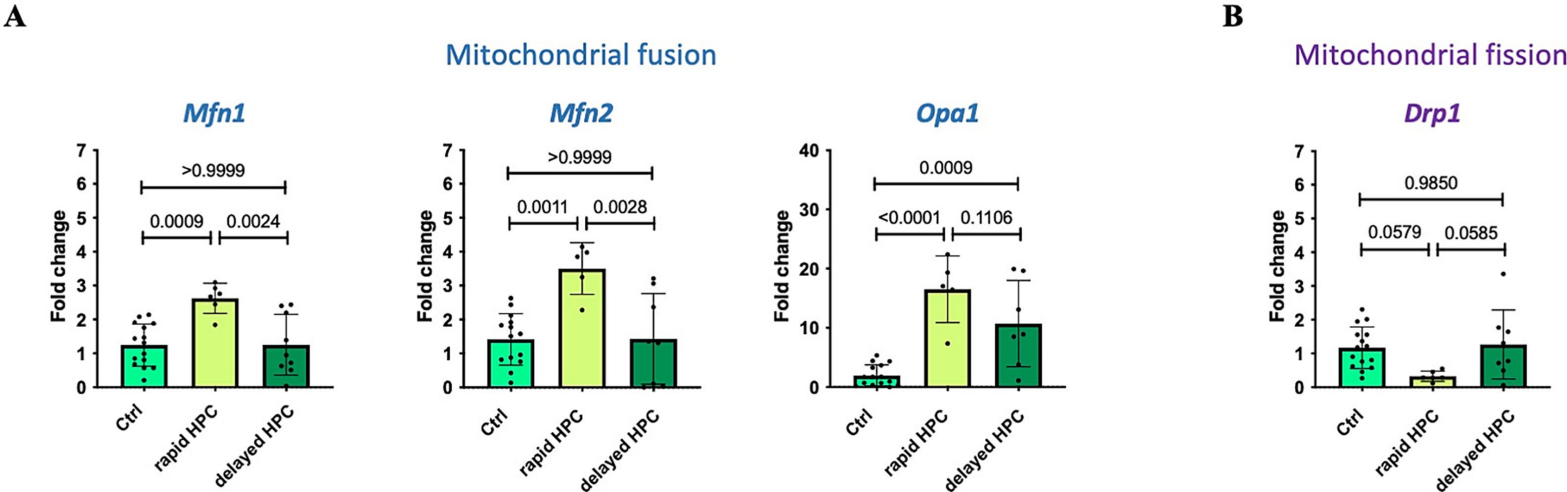
Effect of rapid and delayed HPC-treatment on mRNA expression levels of critical mitochondrial fusion **(A)** and fission **(B)** factors. Real-time qPCR analysis was used to assess the mRNA expression levels of specific target genes, such as fusion factors *Mf1*, *Mfn2*, and *Opa1*, as well as the fission factor *Drp1*. Three distinct mouse groups were examined: untreated (Ctrl), immediately after hypoxia (rapid HPC), and 17 h after hypoxia (delayed HPC). The expression levels were normalized to a housekeeping gene (*Actb*) and are reported as fold changes relative to the Ctrl group. Data represent means ± SD, and statistical significance was determined through one-way ANOVA, with a significance threshold of ≤ 0.05. 15 control and 6 rapid HPC and 8 HPC treated mice were analysed. For *Mfn2* one control and one rapid HPC treated mouse was identified and excluded from analysis. For *Opa 1* one rapid HPC and one delayed HPC treated mouse was identified and excluded from analysis.

Real-time qPCR analyses were employed to assess the expression of mitochondrial fusion and fission factors. The outcomes, presented in Figure 7, represent pooled data, combining results for hippocampus, motor cortex, and striatum. Supplementary Figure S11 provides an overview of the mRNA expression in individual brain regions. Fifteen control and 6 rapid HPC and 8 delayed HPC treated mice were analysed. As for both treatment groups control animals were included in each analytical run, the N of controls is higher than the N in the treatment groups. For *Mfn2* one control and one rapid HPC treated mouse was identified and excluded from analysis. For *Opa 1* one rapid HPC and one delayed HPC treated mouse was identified and excluded from analysis.

Subsequent to hypoxia treatment (rapid HPC group), a significant upregulation in the expression of mitochondrial fusion factors (*Mfn1*, *Mfn2*, and *Opa1*) was noted. However, after a 17-h interval (delayed HPC group), the expression levels of *Mfn1* and *Mfn2* exhibited no significant differences compared to control levels; conversely, these levels were significantly reduced in comparison to the rapid HPC group. Meanwhile, *Opa1* expression also displayed a significant increase in the delayed HPC group compared to the Ctrl group.

While the expression of the fission factor *Drp1* did not exhibit significant changes between the groups overall, a closer examination of individual expression levels revealed a significant reduction in *Drp1* expression immediately after hypoxia in hippocampal and motor cortical tissue, although such reduction was not observed in striatal tissue (Supplementary Figure S11). The expression of the reference gene beta-actin was not changed under the mild hypoxic conditions. Ct values were 19.99 ± 0,71 (*N* = 41) for naïve mice; 20,27 ± 0,82 (*N* = 21) for the delayed HPC group and 20,01 ± 0,63 (*N* = 13) for the immediately analyzed group.

#### 3.3.2 Microscopy analysis

To gain a first impression of morphological changes induced by HPC we exposed CHO cells to HPC. Cultured cells in a monolayer provide a much cleared picture of mitochondrial morphology than the complex situation in slices from mouse brain. Fluorescence microscopy was employed to capture images of stained mitochondria in CHO cells, as illustrated in Figure 3A. Notably, in the rapid HPC group, mitochondria exhibited heightened fusion, resulting in elongated interconnected structures compared to Ctrl conditions. While some mitochondria in the delayed HPC group displayed an increase in fusion, most of them exhibited a Ctrl-like phenotype. The analyses of the images (Figure 3C) indicate a significant rise in the mean mitochondrial area and a significant reduction in the number of mitochondria per cell area immediately after hypoxia. In contrast, the delayed HPC group exhibited no differences compared to the Ctrl group concerning both the mean mitochondrial area and the number of mitochondria per cell area. Additionally, the total mitochondrial area per cell area remained unaffected by the hypoxia treatment across the tested groups.

## 4 Discussion

Hypoxia, characterized by an insufficient O_2_ supply to tissues, poses a substantial threat to the central nervous system. The brain, heavily reliant on O_2_ for its energy demands, is particularly susceptible to hypoxic challenges (Carreau et al., 2011). Nonetheless, depending on the duration and intensity of hypoxia, cells have the capacity to adapt, experience damage, or undergo cell death (Schonenberger and Kovacs, 2015), underscoring the importance of controlled hypoxia in determining the outcome. Mild oxygen deficiency has the potential to induce cellular stress, culminating in a protective or tolerant state referred to as preconditioning (Stetler et al., 2014). HPC entails the application of mild and transient hypoxia, triggering cellular protection and enhancing the cells’ capacity to endure fluctuating O_2_ levels (Li et al., 2017). Several studies demonstrated the beneficial outcomes of HPC on various neurodegenerative diseases including seizures and epilepsy (Gao et al., 2014; Yang et al., 2013; Pohle and Rauca, 1994). Despite the potential of hypoxic preconditioning as a future treatment approach for neurodegenerative diseases, the field still remains understudied. Consequently, a deeper understanding of the mechanisms triggered by mild hypoxia treatment, as these mechanisms could be involved in the neuroprotective benefits, is essential to pave the way for the development of future treatment possibilities. This study aimed to further explore the biochemical changes triggered by mild hypoxia, with a particular emphasis on mitochondria, the central hub of O_2_ consumption.

### 4.1 Seizure threshold

To investigate the neuroprotective potential of the used HPC protocol, we assessed its impact on the seizure threshold using the acute PTZ seizure mouse model. Our results demonstrated a positive influence on the seizure threshold after HPC. This observation is consistent with the beneficial outcomes of HPC on seizures and epilepsy documented in various studies (Gao et al., 2014, Yang et al., 2013, Pohle and Rauca, 1994).

### 4.2 Mitochondrial respiration

To explore the effects of varying O_2_ levels on mitochondrial respiration, high-resolution respirometry experiments were performed under both air-saturated O_2_ conditions and O_2_ levels more aligned with physiological conditions for brain tissue cells (Hadanny and Efrati, 2020). Interestingly, our investigation of mitochondrial respiration under different O_2_ levels revealed diverse results. When measurements were conducted near air-saturated O_2_ levels, we observed a decrease in N-linked LEAK respiration and S-linked ET capacity following HPC treatment compared to the Ctrl group (mass-specific O_2_ flux, CS-specific O_2_ flux). Moreover, the CS-specific O_2_ flux for N- and NS-linked OXPHOS, along with NS-linked ET capacity, also exhibited significant reductions after hypoxia treatment in comparison to the Ctrl group. Taken together, these results indicate that evaluating mitochondrial respiration following HPC near air-saturated O_2_ conditions leads to a reduction in O_2_ consumption compared to Ctrl conditions, with notable effects observed particularly on N-linked LEAK and S-linked ET capacity.

However, when measurements were conducted at O_2_ levels closer to intracellular tissue normoxia, both mass-specific and CS-specific O_2_ flux across all tested states exhibited a significant increase following HPC treatment in comparison to the Ctrl group. Additionally, the *FCR* of N-linked LEAK respiration displayed a significant increase in comparison to the Ctrl group. The observed elevation in mass-specific O_2_ flux post-HPC, along with unchanged *FCR* values during OXPHOS and ET capacity but an augmentation in O_2_ flux and *FCR* during LEAK respiration, implies HPC treatment triggers mt-quality changes which may be attributed to a rise in LEAK respiration. This interpretation gains further support from the observed decreased *P*-*L* control efficiency post-hypoxia, indicative of a less tightly coupled system (Gnaiger et al., 2020; Gnaiger, 2020).

These results collectively indicate that Ctrl and HPC-treated mitochondria exhibit distinct responses to experimental O_2_ conditions. The main observation is a direct dependence of respiration on oxygen concentrations in controls. By contrast, HPC treated mice do not display markedly reduced respiration at tissue normoxic conditions as compared to air saturated conditions.

### 4.3 Mitochondrial ROS production

Our findings demonstrated an elevated CS-specific H_2_O_2_ flux during the N-linked LEAK state following HPC. Additionally, H_2_O_2_ production significantly increased, particularly during N-linked OXPHOS (mass-specific and CS-specific H_2_O_2_ flux as well as *J*_H2O2_/*J*_O2_). Moreover, mass-specific H_2_O_2_ flux and the *J*_H2O2_/*J*_O2_ ratio, both assessed after adding Ama to determine the maximal contribution of CIII to ROS production, exhibited a significant decrease following hypoxia treatment. These observations suggest increased ROS production primarily originating from CI, while the contribution of CIII to ROS generation appears to be diminished. Furthermore, the observed rise in O_2_ consumption after HPC under intracellular tissue normoxia may be partly attributed to the increased generation of ROS compared to the control.

Mitochondrial ROS production under hypoxic conditions has a dual impact. On one hand, it can lead to cellular damage, while on the other hand, it can activate signaling pathways (Azzi, 2022). After a hypoxic phase when the ETS is highly reduced and O_2_ levels rise again, ROS production can be stimulated, causing oxidative tissue damage (Qin et al., 2009). Current evidence points to CI as the source of increased ROS production contributing to tissue injuries (Brand et al., 2016; Gorenkova et al., 2013). However, in our experiments, it would be intriguing to pinpoint the specific ROS production site within CI. CI has two known ROS production sites: I_Q_, believed to be active during RET at the ubiquinone binding site, and I_F_, located at the flavin site of CI and suggested to be active during forward electron transfer (Gibbs et al., 2023; Brand et al., 2016). The I_Q_ site has been associated with ischemia–reperfusion damage, and experiments blocking ROS production specifically from this site, without inhibiting respiration or OXPHOS, have shown protective effects against ischemia–reperfusion injuries when RET was responsible for ROS generation (Brand et al., 2016; Yin et al., 2021).

However, we noticed that the observed rise in ROS production corresponds to less than 2% (*J*_H2O2_/*J*_O2_*100) of the consumed O_2_. This falls within the range of ROS production observed under physiological conditions, where it is estimated to be as high as 2% of the consumed O_2_ (Zorov et al., 2014). As concluded from our mitochondrial morphology and dynamics study, the measured ROS production seems to remain below the threshold that causes damage, indicating that the slight increase in ROS levels might function as a mechanism for cell signaling and protection. Furthermore, this concept closely resembles the principle of preconditioning, where cells and tissues are exposed to a non-lethal stressor, whether of the same or different origin, leading to cellular and tissue protection (Li et al., 2017; Stetler et al., 2014).

Interestingly, our study suggests that HPC triggers controlled ROS production via CI, maintaining ROS levels below the toxic threshold. This controlled ROS generation could potentially activate various pathways, such as the HIF1 and ERK pro-survival pathways, contributing to the preconditioning effects (Liu et al., 2005; Fryer et al., 2001).

The finding that CI is the primary source of increased ROS production, with a reduced role for CIII, contradicts findings from other studies that suggested CIII as the main ROS source for cell signaling (Bell et al., 2007). For instance, it is argued that ROS originating from CIII theoretically has a more direct path to the cytosol, where they engage in cell signaling (Diebold and Chandel, 2016). Moreover, there is a proposition that actually the O_2_^•-^ derived from CIII possesses the cellular signaling and cytoprotective effects, providing additional support for the significance of CIII in this context (Malinska et al., 2010).

In an intriguing study conducted by Guzy et al. (2005), these authors observed the crucial role of ROS in activating the HIF1 pathway. However, they propose that HIF1α stabilization is predominantly attributed to H_2_O_2_ originating from CIII, a notion supported by the fact that blocking CIII abolishes this stabilizing effect. Nevertheless, it is important to highlight that their discussion recognizes the possibility that interfering with CIII may also perturb the electron flow in CI and CII, casting uncertainty on the involvement of these complexes. Furthermore, the same study emphasized that O_2_^•-^ is not directly necessary for HIF1α stabilization, highlighting the importance of H_2_O_2_ instead (Guzy et al., 2005).

In line with the study of Guzy et al. (2005), the prevailing consensus continues to recognize H_2_O_2_ as the primary ROS involved in cell signaling (Diebold and Chandel, 2016). For instance, elevated H_2_O_2_ levels can induce HIF1α accumulation, even under air-saturated O_2_ conditions (Richard et al., 2000; Chandel et al., 2000), and that the administration of a moderate dose of H_2_O_2_ to cells before subjecting them to ischemic conditions can lead to preconditioning (Lebuffe et al., 2003). Furthermore, CI inhibition and the reduction of mitochondrial ROS levels in the cell culture can effectively prevent HIF1α accumulation, even under conditions with 1.5% O_2_ (Chandel et al., 2000; Agani et al., 2000).

Taken together, these studies support the assumption that H_2_O_2_ originating from CI might be released from the mitochondrial matrix and might play a role in the activation of various cell signaling processes, including the HIF pathway.

The significant disparities observed across various studies regarding the role of ROS in cell signaling, such as the activation of the HIF1 pathway, may be attributed to differences in experimental conditions. These discrepancies encompass the utilization of distinct cell types or tissues, including cancer cell lines, which may respond differently from physiologically relevant cells. Additionally, *in vitro* experiments might create a more artificial environment. The techniques employed for measuring ROS production may also introduce variations in outcomes. Our objective was to tackle these issues by subjecting mice to treatments, enabling us to investigate the impact of HPC on the organism instead of focusing solely on individual cells. Furthermore, we focused on precisely measuring mitochondrial respiration and ROS production while assessing the involvement of various respiratory complexes at a high resolution.

Importantly, our measurements were conducted 17 h post-hypoxia rather than during or immediately after the hypoxic phase. This time point variation may contribute to the discrepancies in results compared to findings in other publications. However, the regulation of mitochondrial ROS generation *in vivo* is still a relatively underexplored area, given that a substantial portion of our current insights has been gleaned from investigations conducted on isolated mitochondria or cells *in vitro*. Numerous factors, including O_2_ levels, electron availability for the ETS, the quantity of electron carriers, the redox state of these carriers, and the mitochondrial membrane potential, can all exert an influence on the generation of mitochondrial ROS (Diebold and Chandel, 2016).

To the best of our knowledge, this study represents the first attempt, to employ high-resolution respirometry for evaluating mitochondrial respiration and ROS production in brain tissue homogenates following *in vivo* exposure to hypoxia.

### 4.4 Mitochondrial dynamics

The evaluation of mitochondrial dynamics following HPC treatment demonstrated an elevation in the mRNA expression levels of fusion factors *Mfn1*, *Mfn2*, and *Opa1* within the rapid HPC group. This observation is in line with reported augmentation in mitochondrial fusion immediately after hypoxia (Macdonald et al., 2016; Akepati et al., 2008). This notion is supported by microscopy images depicting heightened mitochondrial fusion immediately after hypoxia. Additionally, the analysis of mitochondrial morphology demonstrated an elevated average mitochondrial size coupled with a reduction in mitochondrial number per cell area, further bolstering the inference of increased mitochondrial fusion immediately post-hypoxia.

Increased levels of both MFN1 and MFN2 have also been linked to the perinuclear aggregation of mitochondria, a phenomenon associated with the localized release of second messengers, including ROS, towards the nucleus. For instance, these effects have been demonstrated to play a crucial role in the complete activation of HIF1 target genes (Al-Mehdi et al., 2012). The rise in *Mfn1* and *Mfn2* mRNA levels post-hypoxia could potentially lead to augmented perinuclear mitochondrial accumulation, influencing processes such as the activation of HIF1 target genes.

OPA1 not only participates in the fusion of the mitochondrial inner membrane but also plays essential roles in functions like *cristae* morphology and the maintenance of mitochondrial DNA. Moreover, it contributes to reinforcing connections within the *cristae*, which in turn aids in sequestering cytochrome *c* and influencing apoptosis (Del Dotto et al., 2017; Olichon et al., 2003; Frezza et al., 2006). Therefore, the upregulation of *Opa1* expression following hypoxia treatment has the potential to enhance cell survival.

Seventeen hours after hypoxia, mRNA levels of *Mfn1* and *Mfn2* did not exhibit significant changes, while the expression of the fusion factor *Opa1* showed a statistically significant increase compared to Ctrl samples. Notably, microscopy images of stained mitochondria at the same time point revealed mixed results—some cells exhibited heightened mitochondrial fusion, while the majority displayed a mitochondrial fusion level similar to the Ctrl. Additionally, analyses of mitochondrial morphology showed no alterations in the mean mitochondrial size and mitochondrial number per cell area when compared to the Ctrl group. The observed increase in *Opa1* expression 17 h after hypoxia might trigger survival signaling. However, it could also contribute to mitochondrial fusion, potentially explaining the presence of fused mitochondria in some cells.

Additionally, we did not detect any changes in the mitochondrial area relative to the cell area both immediately after hypoxia and 17 h later. This suggests that, under the conditions employed, there was no biogenesis of new mitochondria.

In summary, the findings suggest an immediate increase in mitochondrial fusion following hypoxia, potentially being part of an adaptive process often associated with cell survival (Park et al., 2014). However, 17 h after hypoxia, the majority of cells contained mitochondria with morphology similar to those under control conditions, although some cells displayed heightened mitochondrial fusion. Interestingly, there were no indications of increased mitochondrial fission, typically linked to elevated stress levels, mitochondrial dysfunction, and cell death (Tilokani et al., 2018). This suggests that our treatment did not evoke severe cellular stress resulting in tissue damage or cell death; instead, it remained below the threshold of harmful hypoxia. Enhanced mitochondrial fusion is frequently linked to mild stress responses and associated with mechanisms that promote cell survival, protecting against mitochondrial degradation (Rambold et al., 2011). This kind of mild stress-triggered hyperfusion of mitochondria is believed to be regulated, among other factors, by OPA1 and MFN1 (Tondera et al., 2009). Nonetheless, it is important to acknowledge that although we did observe heightened mitochondrial fusion following hypoxia, only a subset of cells displayed an elevated mitochondrial fusion phenotype 17 h later. This implies that in our experimental configuration, mitochondrial fusion might have a limited role in the neuroprotective effects observed hours after HPC treatment.

## 5 Conclusion

In this study, we were able to show the critical role of O_2_ levels during mitochondrial respiration measurements, as changes in O_2_ levels can significantly influence the results and potentially lead to misleading conclusions. In our particular context, it is crucial to perform respiration measurements under O_2_ levels that closely resemble physiological conditions since our primary objective is to gain insights into how cells and mitochondria adapt to varying O_2_ levels. Remarkably, when hypoxia-exposed mitochondria were subjected to air-saturated, effectively hyperoxic conditions, they tended to exhibit reduced O_2_ consumption compared to control samples. Conversely, under O_2_ levels closer to intracellular tissue normoxia, their O_2_ consumption rate increased when compared to the control. This elevated O_2_ consumption appeared to be associated with an increase in the production of ROS, which remained within the physiological range and primarily emanated from CI, while the contribution of CIII appeared to be diminished in this context. We speculate that this heightened ROS production might play a role in initiating cell signaling, such as the activation of the HIF1 pathway, even in the presence of physiological O_2_ concentrations.

Although we did observe an increase in mitochondrial fusion after hypoxia, this effect was only noticeable in a subset of cells 17 h later. This suggests that, within the context of our experimental setup, mitochondrial fusion may play a minor role in the neuroprotective effects observed hours after HPC treatment. The observed increase in *Mfn1* and *Mfn2* levels might have resulted in a greater aggregation of mitochondria around the nucleus, and when combined with a controlled release of ROS towards the nucleus, it would have the potential to amplify the activation of HIF1 target genes. This collaborative effect could promote pro-survival and neuroprotective signaling, possibly also supported by *Opa1*.

While we did observe an increased seizure threshold, indicating enhanced resistance to epileptic seizures and highlighting the neuroprotective effects of applied HPC protocol, our present results do not allow us to definitively establish a direct connection between the observed post-HPC changes and the protective effects noted.

The results of this study offer insights into the mechanisms that underlie hypoxic preconditioning. These insights have the potential to improve our comprehension of events following mild hypoxia exposure, which could play a role in the neuroprotective and pro-survival effects associated with HPC.

## 6 Outlook and limitations

In future experiments, it will be vital to explore whether the observed rise in ROS production originating from CI can indeed initiate cellular signaling, such as activating the HIF1 pathway, in response to hypoxia exposure. Additionally, it would be important to pinpoint the exact site within CI responsible for ROS generation and its role in protective effects by employing specific CI inhibitors.

Furthermore, we assessed mitochondrial respiration in tissue samples from the hippocampus, motor cortex, and striatum near air-saturated O_2_ conditions. However, in measurements under conditions of intracellular tissue normoxia, we were only able to conduct experiments using samples from the hippocampus and striatum. This limitation arose because we utilized the same samples to measure ROS production, and our capacity to perform measurements on all three tissue samples simultaneously was constrained due to a limited number of Oroboros devices.

Moreover, although the Macro tool we created simplifies the analysis of mitochondrial morphology, it still requires manual adjustments to correct false positives.

## Data Availability

The original contributions presented in the study are included in the article/Supplementary material, further inquiries can be directed to the corresponding author.

## Supplementary material

SUPPLEMENTARY FIGURE S1 Mitochondrial respiration and H_2_O_2_ production assessed under tissue normoxia (O_2_ concentration 30 to 40 μM). Representative traces of mitochondrial respiration **(A)** and H_2_O_2_ production **(B)** simultaneously measured by high-resolution respirometry (Oroboros FluoRespirometer). The experimental protocol closely matched the SUIT protocol in Figure 2 (main text). 1PMG: Sequential injections of pyruvate (P; 5 mM), malate (M; 2 mM), and glutamate (G; 10 mM) to initiate N-linked LEAK respiration N*_L_*. 2D: A kinetically saturating ADP concentration (D; 2.5 mM) induced N-linked OXPHOS N*_P_*. 3S: CII-substrate succinate (S; 50 mM) added to evaluate NS-pathway OXPHOS capacity NS*_P_* (CI- and CII-linked). 4U: Stepwise titrations of the uncoupler carbonyl cyanide chlorophenylhydrazone CCCP (U; 1.5–2.5 μM) to reach maximum respiration as an estimate of NS-pathway ET capacity NS*_E_*. 5Rot: Inhibition of CI by rotenone (Rot; 0.5 μM) to measure S-linked ET capacity S*_E_*. 6Ama: Inhibition of CIII by antimycin A (Ama; 2.5 μM) to assess residual oxygen consumption *Rox* for baseline correction of O_2_ flux in all mitochondrial respiratory states. During sections of reoxygenations, marked by shaded bars, fluxes cannot be measured. **(A)** The blue line shows O_2_ concentration [μM] with a zoom into the 50 μM range (compare Figure 2), the red line shows O_2_ flux per tissue wet mass [pmol_·_s^−1^_·_mg^−1^]. Marks (vertical bars) for evaluation of rates were set at O_2_ concentrations of 30 to 40 μM. O_2_ levels were adjusted by introducing N_2_ or H_2_ or opening the chambers. **(B)** Fluorometric signal (black line), calculated as equivalent H_2_O_2_ concentration [μM] (note that the actual H_2_O_2_ concentration is maintained at zero in the AmR assay). The green line denotes H_2_O_2_ flux per tissue mass [pmol_·_s^−1^_·_mg^−1^]. Changes of fluorescence sensitivity over time were assessed by sequentially titrating H_2_O_2_ (0.1 μM) in the course of the experiment. The black bar indicates the maximal contribution of CIII to ROS production. Titration spikes are disturbances of the traces of O_2_ flux apparent as downwards spikes and of H_2_O_2_ flux apparent as upwards spikes.

SUPPLEMENTARY FIGURE S2 Effect of AmR on mitochondrial respiration in brain tissue of untreated mice assessed under tissue normoxia. Mitochondrial respiration measured at O_2_ concentrations ranging from 30-40 μM, either in the absence of the H_2_O_2_ fluorescence probe (w/o AmR) or its presence (AmR+fluorescence light). See legend of Supplementary Figure S1 for respiratory states and rates. Mass-specific O_2_ flux [pmol_·_s^−1^_·_mg^−1^] was normalized for wet tissue mass. Citrates synthase activity (CS [IU]) was used as a mitochondrial marker for expression of mt-specific O_2_ flux [pmol_·_s^−1^_·_IU^−1^]. Flux control ratios *FCR* were normalized for maximal O_2_ flux NS*_E_*. Samples were harvested from the hippocampus and striatum of untreated C57BL6/J mice. Data represent medians ± IQR and statistical significance was determined using the Mann-Whitney U test, with a significance level set at ≤ 0.05.

SUPPLEMENTARY FIGURE S3 Effect of AmR on mitochondrial respiration of hippocampus and striatum from HPC treated mice assessed under tissue normoxia. Mitochondrial respiration measured at O_2_ concentrations of 30-40 μM, either in the absence of the H_2_O_2_ fluorescence probe (w/o AmR) or its presence (AmR+fluorescence light). See legend of Supplementary Figure S1 for respiratory states and rates, and legend of Supplementary Figure S2 for normalization of O_2_ flux. Data represent medians ± IQR and statistical significance was determined using the Mann-Whitney U test, with a significance level set at ≤ 0.05.

SUPPLEMENTARY FIGURE S4 Effect of AmR on respiratory control efficiencies in controls and HPC-treated mice in hippocampus and striatum assessed under tissue normoxia. Control ratios in the presence or absence of H_2_O_2_ fluorescence probe, AmR. *P*-*L* control efficiency = (N*_P_*−N*_L_*)/N*_P_* = 1−N*_L_*/N*_P_*; *E*-*P* control efficiency = (NS*_E_*−NS*_P_*)/NS*_E_* = 1−NS*_P_*/NS*_E_*. See legend of Supplementary Figure S1 for respiratory states and rates. Data represent medians ± IQR. Statistical test: Mann-Whitney U test, ≤ 0.05, was defined as statistically significant.

SUPPLEMENTARY FIGURE S5 Effect of HPC-treatment on mitochondrial respiration of different brain areas assessed under effectively hyperoxic conditions near air-saturation. Homogenates were prepared from hippocampus, motor cortex, and striatum of Ctrl and HPC-treated C57BL6/J mice. See legend of Supplementary Figure S1 for respiratory states and rates, and legend of Supplementary Figure S2 for normalization of O_2_ flux. Data represent medians ± IQR. Statistical test: Mann-Whitney U test, ≤ 0.05, was defined as statistically significant.

SUPPLEMENTARY FIGURE S6 Effect of HPC-treatment on respiratory control efficiencies in different brain areas assessed under effectively hyperoxic conditions near air-saturation. See legend of Supplementary Figure S1 for respiratory states and rates, and legend of Supplementary Figure S4 for respiratory control efficiencies. Data represent medians ± IQR. Statistical test: Mann-Whitney U test, ≤ 0.05, was defined as statistically significant.

SUPPLEMENTARY FIGURE S7 Effect of HPC-treatment on mitochondrial respiration of hippocampus and striatum assessed under intracellular tissue normoxia. Tissue homogenates of Ctrl and HPC-treated C57BL6/J mice were examined under O_2_ concentrations of 30–40 μM. See legend of Supplementary Figure S1 for respiratory states and rates, and legend of Supplementary Figure S2 for normalization of O_2_ flux. Data represent medians ± IQR. Statistical test: Mann-Whitney U test, ≤ 0.05, was defined as statistically significant.

SUPPLEMENTARY FIGURE S8 Effect of HPC-treatment on respiratory control efficiencies of hippocampus and striatum assessed under intracellular tissue normoxia. Measurements on brain homogenates of Ctrl and HPC-treated C57BL6/J mice performed under O_2_ concentrations of 30–40 μM. See legend of Supplementary Figure S1 for respiratory states and rates, and legend of Supplementary Figure S4 for respiratory control efficiencies. Data represent medians ± IQR. Statistical test: Mann-Whitney U test, ≤ 0.05, was defined as statistically significant.

SUPPLEMENTARY FIGURE S9 Effect of HPC-treatment on net mitochondrial H_2_O_2_ production of hippocampus and striatum assessed under tissue normoxia. Measurements on tissue homogenates from Ctrl and HPC-treated C57BL6/J mice were conducted while maintaining O_2_ concentration within the range of 30–40 μM. The *J*_H2O2_/*J*_O2_ ratio is the proportion of H_2_O_2_ produced to O_2_ consumed. H_2_O_2_ flux was normalized to O_2_ flux in the absence of AmR. See legend of Supplementary Figure S1 for respiratory states and rates, and legend of Supplementary Figure S2 for normalization of H_2_O_2_ flux. Data represent medians ± IQR. Statistical test: Mann-Whitney U test, ≤ 0.05, was defined as statistically significant.

SUPPLEMENTARY FIGURE S10 Effect of HPC-treatment on maximal contribution of CIII to mitochondrial H_2_O_2_ production in hippocampus and striatum under tissue normoxia. The fluorescence assay AmR was employed to determine H_2_O_2_ flux. The maximal contribution of CIII to the net production of H_2_O_2_ was measured in tissue homogenates from control and HPC-treated C57BL6/J mice, using the CIII inhibitor antimycin A at O_2_ concentrations of 30–40 μM. See legend of Supplementary Figure S1 for respiratory states and rates, and legend of Supplementary Figures S2, S9 for normalization of H_2_O_2_ flux. Data represent medians ± IQR. Statistical test: Mann-Whitney U test, ≤ 0.05, was defined as statistically significant.

SUPPLEMENTARY FIGURE S11 Effect of rapid and delayed HPC-treatment on mRNA expression of critical factors associated with mitochondrial fusion and fission in different brain areas. We conducted real-time qPCR analyses to examine the mRNA expression levels of various target genes in three distinct groups: Control, rapid HPC (samples collected immediately after hypoxia) and delayed HPC (samples obtained 17 hours after hypoxia) in C57BL6/J mice. These assessments were carried out on tissues from the hippocampus, motor cortex, and striatum. The target genes under investigation included fusion factors *Mfn1*, *Mfn2*, and *Opa1*, as well as the fission factor *Drp1*. Expression levels were standardized by comparing them to the expression of a housekeeping gene (*Actb*) and are reported as fold changes in relation to the control. Data represent means ± SD and statistical significance was determined using the one-way ANOVA test, with a significance threshold set at ≤ 0.05.

## References

[ref1] Agani F. H. Pichiule P. Chavez J. C. Lamanna J. C. (2000). The role of mitochondria in the regulation of hypoxia-inducible factor 1 expression during hypoxia. J. Biol. Chem. 275, 35863–35867. doi: 10.1074/jbc.M005643200, PMID: 10961998

[ref2] Akepati V. R. Muller E. C. Otto A. Strauss H. M. Portwich M. Alexander C. (2008). Characterization of OPA1 isoforms isolated from mouse tissues. J. Neurochem. 106, 372–383. doi: 10.1111/j.1471-4159.2008.05401.x, PMID: 18419770

[ref3] Al-Mehdi A. B. Pastukh V. M. Swiger B. M. Reed D. J. Patel M. R. Bardwell G. C. . (2012). Perinuclear mitochondrial clustering creates an oxidant-rich nuclear domain required for hypoxia-induced transcription. Sci. Signal. 5:ra47. doi: 10.1126/scisignal.2002712, PMID: 22763339 PMC3565837

[ref4] Azzi A. (2022). Oxidative stress: what is it?. Can it be measured? Where is it located? Can it be good or bad? Can it be prevented? Can it be cured? Antioxidants 11:1431. doi: 10.3390/antiox11081431, PMID: 35892633 PMC9329886

[ref5] Barone F. C. White R. F. Spera P. A. Ellison J. Currie R. W. Wang X. . (1998). Ischemic preconditioning and brain tolerance: temporal histological and functional outcomes, protein synthesis requirement, and interleukin-1 receptor antagonist and early gene expression. Stroke 29, 1937–1951. doi: 10.1161/01.STR.29.9.1937, PMID: 9731622

[ref6] Bell E. L. Klimova T. A. Eisenbart J. Moraes C. T. Murphy M. P. Budinger G. R. S. . (2007). The Q(o) site of the mitochondrial complex III is required for the transduction of hypoxic signaling via reactive oxygen species production. J. Cell Biol. 177, 1029–1036. doi: 10.1083/jcb.200609074, PMID: 17562787 PMC2064363

[ref7] Bergmeister L. (2023) Functional and molecular aspects of hypoxic preconditioning. Innsbruck, Austria: Ph.D, Medical University of Innsbruck.

[ref8] Bhatti J. Nascimento B. Akhtar U. Rhind S. G. Tien H. Nathens A. . (2018). Systematic review of human and animal studies examining the efficacy and safety of N-acetylcysteine (NAC) and N-acetylcysteine amide (NACA) in traumatic brain injury: impact on neurofunctional outcome and biomarkers of oxidative stress and inflammation. Front. Neurol. 8:744. doi: 10.3389/fneur.2017.00744, PMID: 29387038 PMC5776005

[ref9] Bleier L. Wittig I. Heide H. Steger M. Brandt U. Drose S. (2015). Generator-specific targets of mitochondrial reactive oxygen species. Free Radic. Biol. Med. 78, 1–10. doi: 10.1016/j.freeradbiomed.2014.10.511, PMID: 25451644

[ref10] Brand M. D. (2010). The sites and topology of mitochondrial superoxide production. Exp. Gerontol. 45, 466–472. doi: 10.1016/j.exger.2010.01.003, PMID: 20064600 PMC2879443

[ref11] Brand M. D. (2016). Mitochondrial generation of superoxide and hydrogen peroxide as the source of mitochondrial redox signaling. Free Radic. Biol. Med. 100, 14–31. doi: 10.1016/j.freeradbiomed.2016.04.001, PMID: 27085844

[ref12] Brand M. D. Goncalves R. L. Orr A. L. Vargas L. Gerencser A. A. Borch Jensen M. . (2016). Suppressors of superoxide-H(2)O(2) production at site I(Q) of mitochondrial complex I protect against stem cell hyperplasia and ischemia-reperfusion injury. Cell Metab. 24, 582–592. doi: 10.1016/j.cmet.2016.08.012, PMID: 27667666 PMC5061631

[ref13] Burtscher J. Bean C. Zangrandi L. Kmiec I. Agostinho A. Scorrano L. . (2018). Proenkephalin derived peptides are involved in the modulation of mitochondrial respiratory control during epileptogenesis. Front. Mol. Neurosci. 11:351. doi: 10.3389/fnmol.2018.00351, PMID: 30319356 PMC6167428

[ref14] Burtscher J. Mallet R. T. Burtscher M. Millet G. P. (2021). Hypoxia and brain aging: neurodegeneration or neuroprotection? Ageing Res. Rev. 68:101343. doi: 10.1016/j.arr.2021.101343, PMID: 33862277

[ref15] Burtscher J. Zangrandi L. Schwarzer C. Gnaiger E. (2015). Differences in mitochondrial function in homogenated samples from healthy and epileptic specific brain tissues revealed by high-resolution respirometry. Mitochondrion 25, 104–112. doi: 10.1016/j.mito.2015.10.007, PMID: 26516105

[ref16] Buttgereit F. Brand M. D. (1995). A hierarchy of ATP-consuming processes in mammalian-cells. Biochem. J. 312, 163–167. doi: 10.1042/bj3120163 7492307 PMC1136240

[ref17] Carreau A. El Hafny-Rahbi B. Matejuk A. Grillon C. Kieda C. (2011). Why is the partial oxygen pressure of human tissues a crucial parameter? Small molecules and hypoxia. J. Cell. Mol. Med. 15, 1239–1253. doi: 10.1111/j.1582-4934.2011.01258.x, PMID: 21251211 PMC4373326

[ref18] Chandel N. S. Mcclintock D. S. Feliciano C. E. Wood T. M. Melendez J. A. Rodriguez A. M. . (2000). Reactive oxygen species generated at mitochondrial complex III stabilize hypoxia-inducible factor-1 alpha during hypoxia - a mechanism of O-2 sensing. J. Biol. Chem. 275, 25130–25138. doi: 10.1074/jbc.M001914200, PMID: 10833514

[ref19] Cheignon C. Tomas M. Bonnefont-Rousselot D. Faller P. Hureau C. Collin F. (2018). Oxidative stress and the amyloid beta peptide in Alzheimer's disease. Redox Biol. 14, 450–464. doi: 10.1016/j.redox.2017.10.014, PMID: 29080524 PMC5680523

[ref20] Chen Z. Brodie M. J. Liew D. Kwan P. (2018). Treatment outcomes in patients with newly diagnosed epilepsy treated with established and new antiepileptic drugs: a 30-year longitudinal cohort study. JAMA Neurol. 75, 279–286. doi: 10.1001/jamaneurol.2017.3949, PMID: 29279892 PMC5885858

[ref21] Curia G. Lucchi C. Vinet J. Gualtieri F. Marinelli C. Torsello A. . (2014). Pathophysiogenesis of mesial temporal lobe epilepsy: is prevention of damage antiepileptogenic? Curr. Med. Chem. 21, 663–688. doi: 10.2174/0929867320666131119152201, PMID: 24251566 PMC4101766

[ref22] Dahl N. A. Balfour W. M. (1964). Prolonged anoxic survival due to anoxia pre-exposure - brain ATP lactate and pyruvate. Am. J. Phys. 207, 452–456. doi: 10.1152/ajplegacy.1964.207.2.452 14205366

[ref23] Del Dotto V. Mishra P. Vidoni S. Fogazza M. Maresca A. Caporali L. . (2017). OPA1 isoforms in the hierarchical organization of mitochondrial functions. Cell Rep. 19, 2557–2571. doi: 10.1016/j.celrep.2017.05.073, PMID: 28636943

[ref24] Diebold L. Chandel N. S. (2016). Mitochondrial ROS regulation of proliferating cells. Free Radic. Biol. Med. 100, 86–93. doi: 10.1016/j.freeradbiomed.2016.04.198, PMID: 27154978

[ref25] Doerrier C. Garcia-Souza L. F. Krumschnabel G. Wohlfarter Y. Meszaros A. T. Gnaiger E. (2018). High-resolution fluoRespirometry and OXPHOS protocols for human cells, permeabilized fibers from small biopsies of muscle, and isolated mitochondria. Methods Mol. Biol. 1782, 31–70. doi: 10.1007/978-1-4939-7831-1_3, PMID: 29850993

[ref26] Donnelly C. Schmitt S. Cecatto C. Cardoso L. Komlódi T. Place N. . (2022). The ABC of hypoxia – what is the norm. Bioenerg. Commun. doi: 10.26124/mitofit:2022-0025.v2

[ref27] Emerson M. R. Nelson S. R. Samson F. E. Pazdernik T. L. (1999). Hypoxia preconditioning attenuates brain edema associated with kainic acid-induced status epilepticus in rats. Brain Res. 825, 189–193. doi: 10.1016/s0006-8993(99)01195-6, PMID: 10216187

[ref28] Fisher R. S. Acevedo C. Arzimanoglou A. Bogacz A. Cross J. H. Elger C. E. . (2014). ILAE official report: a practical clinical definition of epilepsy. Epilepsia 55, 475–482. doi: 10.1111/epi.12550 24730690

[ref29] Frezza C. Cipolat S. Martins De Brito O. Micaroni M. Beznoussenko G. V. Rudka T. . (2006). OPA1 controls apoptotic cristae remodeling independently from mitochondrial fusion. Cell 126, 177–189. doi: 10.1016/j.cell.2006.06.025, PMID: 16839885

[ref30] Fryer R. M. Patel H. H. Hsu A. K. Gross G. J. (2001). Stress-activated protein kinase phosphorylation during cardioprotection in the ischemic myocardium. Am. J. Physiol. Heart Circ. Physiol. 281, H1184–H1192. doi: 10.1152/ajpheart.2001.281.3.H1184 11514286

[ref31] Gao C. J. Niu L. Ren P. C. Wang W. Zhu C. Li Y. Q. . (2012). Hypoxic preconditioning attenuates global cerebral ischemic injury following asphyxial cardiac arrest through regulation of delta opioid receptor system. Neuroscience 202, 352–362. doi: 10.1016/j.neuroscience.2011.11.060, PMID: 22200548

[ref32] Gao C. Wang C. Liu B. Wu H. Yang Q. L. Jin J. G. . (2014). Intermittent hypoxia preconditioning-induced epileptic tolerance by upregulation of monocarboxylate transporter 4 expression in rat hippocampal astrocytes. Neurochem. Res. 39, 2160–2169. doi: 10.1007/s11064-014-1411-2, PMID: 25146899

[ref33] Gibbs E. T. Lerner C. A. Watson M. A. Wong H. S. Gerencser A. A. Brand M. D. (2023). Site IQ in mitochondrial complex I generates S1QEL-sensitive superoxide/hydrogen peroxide in both the reverse and forward reactions. Biochem. J. 480, 363–384. doi: 10.1042/BCJ20220611, PMID: 36862427 PMC10212513

[ref34] Gnaiger E. (1993). Nonequilibrium thermodynamics of energy transformations. Pure Appl. Chem. 65, 1983–2002. doi: 10.1351/pac199365091983

[ref35] Gnaiger E. (2020). Mitochondrial pathways and respiratory control: an introduction to OXPHOS analysis. Bioenerg. Commun. 1–114. doi: 10.26124/bec:2020-0001.v1

[ref36] Gnaiger E. Aasander Frostner E. Abdul K. Abdel-Rahman E. Abumrad N. Acuna-Castroviejo D. . (2020). Mitochondrial physiology. Bioenerg. Commun.:2020.1.

[ref37] Gorenkova N. Robinson E. Grieve D. J. Galkin A. (2013). Conformational change of mitochondrial complex i increases ROS sensitivity during ischemia. Antioxid. Redox Signal. 19, 1459–1468. doi: 10.1089/ars.2012.4698 23419200 PMC3797456

[ref38] Guzy R. D. Hoyos B. Robin E. Chen H. Liu L. P. Mansfield K. D. . (2005). Mitochondrial complex III is required for hypoxia-induced ROS production and cellular oxygen sensing. Cell Metab. 1, 401–408. doi: 10.1016/j.cmet.2005.05.001, PMID: 16054089

[ref39] Hadanny A. Efrati S. (2020). The hyperoxic-hypoxic paradox. Biomolecules 10:958. doi: 10.3390/biom10060958, PMID: 32630465 PMC7355982

[ref40] Hoehne M. N. Jacobs L. J. H. C. Lapacz K. J. Calabrese G. Murschall L. M. Marker T. . (2022). Spatial and temporal control of mitochondrial H_2_O_2_ release in intact human cells. EMBO J. 41:e109169. doi: 10.15252/embj.2021109169, PMID: 35146782 PMC8982624

[ref41] Hou P. Kuo C. Y. Cheng C. T. Liou J. P. Ann D. K. Chen Q. (2014). Intermediary metabolite precursor dimethyl-2-ketoglutarate stabilizes hypoxia-inducible factor-1alpha by inhibiting prolyl-4-hydroxylase PHD2. PLoS One 9:e113865. doi: 10.1371/journal.pone.0113865, PMID: 25420025 PMC4242664

[ref42] Kirino T. (2002). Ischemic tolerance. J. Cereb. Blood Flow Metab. 22, 1283–1296. doi: 10.1097/01.WCB.0000040942.89393.88, PMID: 12439285

[ref43] Komlódi T. Geibl F. F. Sassani M. Ambrus A. Tretter L. (2018a). Membrane potential and delta pH dependency of reverse electron transport-associated hydrogen peroxide production in brain and heart mitochondria. J. Bioenerg. Biomembr. 50, 355–365. doi: 10.1007/s10863-018-9766-8, PMID: 30116920 PMC6209044

[ref44] Komlodi T. Sobotka O. Gnaiger E. (2021). Facts and artefacts on the oxygen dependence of hydrogen peroxide production using Amplex UltraRed. Bioenerg. Commun. doi: 10.26124/bec:2021-0004

[ref45] Komlódi T. Sobotka O. Krumschnabel G. Bezuidenhout N. Hiller E. Doerrier C. . (2018b). Comparison of mitochondrial incubation Media for Measurement of respiration and hydrogen peroxide production. Methods Mol. Biol. 1782, 137–155. doi: 10.1007/978-1-4939-7831-1_8, PMID: 29850998

[ref46] Kopp S. J. Krieglstein J. Freidank A. Rachman A. Seibert A. Cohen M. M. (1984). P-31 nuclear magnetic resonance analysis of brain: II. Effects of oxygen deprivation on isolated perfused and nonperfused rat brain. J. Neurochem. 43, 1716–1731. doi: 10.1111/j.1471-4159.1984.tb06100.x, PMID: 6092545

[ref47] Kudin A. P. Kudina T. A. Seyfried J. Vielhaber S. Beck H. Elger C. E. . (2002). Seizure-dependent modulation of mitochondrial oxidative phosphorylation in rat hippocampus. Eur. J. Neurosci. 15, 1105–1114. doi: 10.1046/j.1460-9568.2002.01947.x, PMID: 11982622

[ref48] Lebuffe G. Schumacker P. T. Shao Z. H. Anderson T. Iwase H. Vanden Hoek T. L. (2003). ROS and NO trigger early preconditioning: relationship to mitochondrial KATP channel. Am. J. Physiol. Heart Circ. Physiol. 284, H299–H308. doi: 10.1152/ajpheart.00706.2002 12388274

[ref49] Lee P. Chandel N. S. Simon M. C. (2020). Cellular adaptation to hypoxia through hypoxia inducible factors and beyond. Nat. Rev. Mol. Cell Biol. 21, 268–283. doi: 10.1038/s41580-020-0227-y, PMID: 32144406 PMC7222024

[ref50] Li S. J. Hafeez A. Noorulla F. Geng X. K. Shao G. Ren C. H. . (2017). Preconditioning in neuroprotection: from hypoxia to ischemia. Prog. Neurobiol. 157, 79–91. doi: 10.1016/j.pneurobio.2017.01.001, PMID: 28110083 PMC5515698

[ref51] Liu J. Gu Y. K. Guo M. Y. Ji X. M. (2021). Neuroprotective effects and mechanisms of ischemic/hypoxic preconditioning on neurological diseases. CNS Neurosci. Ther. 27, 869–882. doi: 10.1111/cns.13642, PMID: 34237192 PMC8265941

[ref52] Liu J. Narasimhan P. Yu F. Chan P. H. (2005). Neuroprotection by hypoxic preconditioning involves oxidative stress-mediated expression of hypoxia-inducible factor and erythropoietin. Stroke 36, 1264–1269. doi: 10.1161/01.STR.0000166180.91042.02, PMID: 15890996

[ref53] Long H. Z. Cheng Y. Zhou Z. W. Luo H. Y. Wen D. D. Gao L. C. (2021). PI3K/AKT signal pathway: a target of natural products in the prevention and treatment of Alzheimer's disease and Parkinson's disease. Front. Pharmacol. 12:648636. doi: 10.3389/fphar.2021.648636, PMID: 33935751 PMC8082498

[ref54] Macdonald P. J. Francy C. A. Stepanyants N. Lehman L. Baglio A. Mears J. A. . (2016). Distinct splice variants of dynamin-related protein 1 differentially utilize mitochondrial fission factor as an effector of cooperative GTPase activity. J. Biol. Chem. 291, 493–507. doi: 10.1074/jbc.M115.680181 26578513 PMC4697187

[ref55] Malinska D. Mirandola S. R. Kunz W. S. (2010). Mitochondrial potassium channels and reactive oxygen species. FEBS Lett. 584, 2043–2048. doi: 10.1016/j.febslet.2010.01.013, PMID: 20080090

[ref56] Matsuda T. Shimizu I. Murata Y. Baba A. (1992). Glucose and oxygen deprivation induces a Ca(2+)- mediated decrease in (Na(+)+K+)- Atpase activity in rat-brain slices. Brain Res. 576, 263–270. doi: 10.1016/0006-8993(92)90689-7, PMID: 1387578

[ref57] Mink J. W. Blumenschine R. J. Adams D. B. (1981). Ratio of central nervous system to body metabolism in vertebrates: its constancy and functional basis. Am. J. Phys. 241, R203–R212. doi: 10.1152/ajpregu.1981.241.3.R203 7282965

[ref58] Muller F. L. Liu Y. H. VAN Remmen H. (2004). Complex III releases superoxide to both sides of the inner mitochondrial membrane. J. Biol. Chem. 279, 49064–49073. doi: 10.1074/jbc.M407715200 15317809

[ref59] Murali Mahadevan H. Hashemiaghdam A. Ashrafi G. Harbauer A. B. (2021). Mitochondria in neuronal health: from energy metabolism to Parkinson's disease. Adv. Biol. (Weinh) 5:e2100663. doi: 10.1002/adbi.202100663, PMID: 34382382

[ref60] Olichon A. Baricault L. Gas N. Guillou E. Valette A. Belenguer P. . (2003). Loss of OPA1 perturbates the mitochondrial inner membrane structure and integrity, leading to cytochrome c release and apoptosis. J. Biol. Chem. 278, 7743–7746. doi: 10.1074/jbc.C200677200, PMID: 12509422

[ref61] Olowe R. Sandouka S. Saadi A. Shekh-Ahmad T. (2020). Approaches for reactive oxygen species and oxidative stress quantification in epilepsy. Antioxidants 9:990. doi: 10.3390/antiox9100990, PMID: 33066477 PMC7602129

[ref62] Ostrowski R. P. Zhang J. H. (2020). The insights into molecular pathways of hypoxia-inducible factor in the brain. J. Neurosci. Res. 98, 57–76. doi: 10.1002/jnr.24366, PMID: 30548473

[ref63] Pan L. N. Zhu W. Li Y. Xu X. L. Guo L. J. Lu Q. . (2014). Astrocytic toll-like receptor 3 is associated with ischemic preconditioning-induced protection against brain ischemia in rodents. PLoS One 9:e99526. doi: 10.1371/journal.pone.0099526, PMID: 24914679 PMC4051824

[ref64] Park Y. Y. Nguyen O. T. Kang H. Cho H. (2014). MARCH5-mediated quality control on acetylated Mfn1 facilitates mitochondrial homeostasis and cell survival. Cell Death Dis. 5:e1172. doi: 10.1038/cddis.2014.142, PMID: 24722297 PMC5424118

[ref65] Pearson-Smith J. N. Patel M. (2017). Metabolic dysfunction and oxidative stress in epilepsy. Int. J. Mol. Sci. 18:2365. doi: 10.3390/ijms18112365, PMID: 29117123 PMC5713334

[ref66] Pereira P. B. A. A. (2016). Quality of life in patients with neurodegenerative diseases. J. Neurol. Neurosci. 7:74. doi: 10.21767/2171-6625.100074

[ref67] Peters A. (2011). The selfish brain: competition for energy resources. Am. J. Hum. Biol. 23, 29–34. doi: 10.1002/ajhb.21106, PMID: 21080380

[ref68] Pohle W. Rauca C. (1994). Hypoxia protects against the neurotoxicity of kainic acid. Brain Res. 644, 297–304. doi: 10.1016/0006-8993(94)91693-4, PMID: 8050040

[ref69] Puspita L. Chung S. Y. Shim J. W. (2017). Oxidative stress and cellular pathologies in Parkinson's disease. Mol. Brain 10:53. doi: 10.1186/s13041-017-0340-9, PMID: 29183391 PMC5706368

[ref70] Qin Y. Jiang W. Li A. Gao M. Liu H. Gao Y. . (2021). The combination of paraformaldehyde and glutaraldehyde is a potential fixative for mitochondria. Biomolecules 11:711. doi: 10.3390/biom11050711, PMID: 34068806 PMC8151741

[ref71] Qin C. Yap S. Woodman O. L. (2009). Antioxidants in the prevention of myocardial ischemia/reperfusion injury. Expert. Rev. Clin. Pharmacol. 2, 673–695. doi: 10.1586/ecp.09.41, PMID: 22112260

[ref72] Rambold A. S. Kostelecky B. Elia N. Lippincott-Schwartz J. (2011). Tubular network formation protects mitochondria from autophagosomal degradation during nutrient starvation. Proc. Natl. Acad. Sci. USA 108, 10190–10195. doi: 10.1073/pnas.1107402108, PMID: 21646527 PMC3121813

[ref73] Rauca C. Zerbe R. Jantze H. Krug M. (2000). The importance of free hydroxyl radicals to hypoxia preconditioning. Brain Res. 868, 147–149. doi: 10.1016/S0006-8993(00)02388-X, PMID: 10841900

[ref74] Reczek C. R. Chandel N. S. (2015). ROS-dependent signal transduction. Curr. Opin. Cell Biol. 33, 8–13. doi: 10.1016/j.ceb.2014.09.010, PMID: 25305438 PMC4380867

[ref75] Redza-Dutordoir M. Averill-Bates D. A. (2016). Activation of apoptosis signalling pathways by reactive oxygen species. Biochim. Biophys. Acta 1863, 2977–2992. doi: 10.1016/j.bbamcr.2016.09.012, PMID: 27646922

[ref76] Richard D. E. Berra E. Pouyssegur J. (2000). Nonhypoxic pathway mediates the induction of hypoxia-inducible factor 1alpha in vascular smooth muscle cells. J. Biol. Chem. 275, 26765–26771. doi: 10.1074/jbc.M003325200 10837481

[ref77] Rogatzki M. J. Ferguson B. S. Goodwin M. L. Gladden L. B. (2015). Lactate is always the end product of glycolysis. Front. Neurosci. 9:22. doi: 10.3389/fnins.2015.00022, PMID: 25774123 PMC4343186

[ref78] Rubaj A. Gustaw K. Zgodziński W. Kleinrok Z. Sieklucka-Dziuba M. (2000). The role of opioid receptors in hypoxic preconditioning against seizures in brain. Pharmacol. Biochem. Behav. 67, 65–70. doi: 10.1016/S0091-3057(00)00294-X 11113485

[ref79] Rybnikova E. Samoilov M. (2015). Current insights into the molecular mechanisms of hypoxic pre- and postconditioning using hypobaric hypoxia. Front. Neurosci. 9:388. doi: 10.3389/fnins.2015.00388, PMID: 26557049 PMC4615940

[ref80] Sanjuan-Pla A. Cervera A. M. Apostolova N. Garcia-Bou R. Victor V. M. Murphy M. P. . (2005). A targeted antioxidant reveals the importance of mitochondrial reactive oxygen species in the hypoxic signaling of HIF-1alpha. FEBS Lett. 579, 2669–2674. doi: 10.1016/j.febslet.2005.03.088 15862307

[ref81] Schonenberger M. J. Kovacs W. J. (2015). Hypoxia signaling pathways: modulators of oxygen-related organelles. Front. Cell Dev. Biol. 3:42. doi: 10.3389/fcell.2015.00042, PMID: 26258123 PMC4508581

[ref82] Scialo F. Fernandez-Ayala D. J. Sanz A. (2017). Role of mitochondrial reverse electron transport in ROS signaling: potential roles in health and disease. Front. Physiol. 8:428. doi: 10.3389/fphys.2017.00428, PMID: 28701960 PMC5486155

[ref83] Semenza G. L. (1999). Regulation of mammalian O_2_ homeostasis by hypoxia-inducible factor 1. Annu. Rev. Cell Dev. Biol. 15, 551–578. doi: 10.1146/annurev.cellbio.15.1.551, PMID: 10611972

[ref84] Shukitt-Hale B. Banderet L. E. Lieberman H. R. (1998). Elevation-dependent symptom, mood, and performance changes produced by exposure to hypobaric hypoxia. Int. J. Aviat. Psychol. 8, 319–334. doi: 10.1207/s15327108ijap0804_1, PMID: 11542275

[ref85] Sies H. (2015). Oxidative stress: a concept in redox biology and medicine. Redox Biol. 4:180-3. doi: 10.1016/j.redox.2015.01.002, PMID: 25588755 PMC4309861

[ref001] Sies H. (2020). Findings in redox biology: From H_2_O_2_ to oxidative stress. J. Biol. Chem. 295, 13458–13473. doi: 10.1074/jbc.X120.015651, PMID: 32978328 PMC7521638

[ref86] Sies H. Jones D. P. (2020). Reactive oxygen species (ROS) as pleiotropic physiological signalling agents. Nat. Rev. Mol. Cell Biol. 21, 363–383. doi: 10.1038/s41580-020-0230-3, PMID: 32231263

[ref87] Silva R. A. D. Peixoto-Santos J. E. Scandiuzzi R. C. Balista P. A. Bassi M. Glass M. L. . (2016). Decreased neuron loss and memory dysfunction in pilocarpine-treated rats pre-exposed to hypoxia. Neuroscience 332, 88–100. doi: 10.1016/j.neuroscience.2016.06.047 27373771

[ref88] Silver I. Erecinska M. (1998). Oxygen and ion concentrations in normoxic and hypoxic brain cells. Adv. Exp. Med. Biol. 454, 7–16. doi: 10.1007/978-1-4615-4863-8_2 9889871

[ref89] Sinenko S. A. Starkova T. Y. Kuzmin A. A. Tomilin A. N. (2021). Physiological signaling functions of reactive oxygen species in stem cells: from flies to man. Front. Cell Dev. Biol. 9:714370. doi: 10.3389/fcell.2021.714370, PMID: 34422833 PMC8377544

[ref90] Stetler R. A. Leak R. K. Gan Y. Li P. Y. Zhang F. Hu X. M. . (2014). Preconditioning provides neuroprotection in models of CNS disease: paradigms and clinical significance. Prog. Neurobiol. 114, 58–83. doi: 10.1016/j.pneurobio.2013.11.005, PMID: 24389580 PMC3937258

[ref91] Tahara E. B. Navarete F. D. T. Kowaltowski A. J. (2009). Tissue-, substrate-, and site-specific characteristics of mitochondrial reactive oxygen species generation. Free Radic. Biol. Med. 46, 1283–1297. doi: 10.1016/j.freeradbiomed.2009.02.008, PMID: 19245829

[ref92] Thijs R. D. Surges R. O'brien T. J. Sander J. W. (2019). Epilepsy in adults. Lancet 393, 689–701. doi: 10.1016/S0140-6736(18)32596-0, PMID: 30686584

[ref93] Thomas L. W. Ashcroft M. (2019). Exploring the molecular interface between hypoxia-inducible factor signalling and mitochondria. Cell. Mol. Life Sci. 76, 1759–1777. doi: 10.1007/s00018-019-03039-y, PMID: 30767037 PMC6453877

[ref94] Thompson J. W. Dave K. R. Young J. I. Perez-Pinzon M. A. (2013). Ischemic preconditioning alters the epigenetic profile of the brain from ischemic intolerance to ischemic tolerance. Neurotherapeutics 10, 789–797. doi: 10.1007/s13311-013-0202-9, PMID: 23868468 PMC3805868

[ref95] Tilokani L. Nagashima S. Paupe V. Prudent J. (2018). Mitochondrial dynamics: overview of molecular mechanisms. Essays Biochem. 62, 341–360. doi: 10.1042/EBC20170104, PMID: 30030364 PMC6056715

[ref96] Tirichen H. Yaigoub H. Xu W. W. Wu C. X. Li R. S. Li Y. F. (2021). Mitochondrial reactive oxygen species and their contribution in chronic kidney disease progression through oxidative stress. Front. Physiol. 12:627837. doi: 10.3389/fphys.2021.627837, PMID: 33967820 PMC8103168

[ref97] Tondera D. Grandemange S. Jourdain A. Karbowski M. Mattenberger Y. Herzig S. . (2009). SLP-2 is required for stress-induced mitochondrial hyperfusion. EMBO J. 28, 1589–1600. doi: 10.1038/emboj.2009.89, PMID: 19360003 PMC2693158

[ref98] Turovskaya M. V. Gaidin S. G. Vedunova M. V. Babaev A. A. Turovsky E. A. (2020). BDNF overexpression enhances the preconditioning effect of brief episodes of hypoxia, promoting survival of GABAergic neurons. Neurosci. Bull. 36, 733–760. doi: 10.1007/s12264-020-00480-z, PMID: 32219700 PMC7340710

[ref99] Van Den Beucken T. Magagnin M. G. Jutten B. Seigneuric R. Lambin P. Koritzinsky M. . (2011). Translational control is a major contributor to hypoxia induced gene expression. Radiother. Oncol. 99, 379–384. doi: 10.1016/j.radonc.2011.05.058 21719133

[ref100] Wang X. L. Su B. Siedlak S. L. Moreira P. I. Fujioka H. Wang Y. . (2008). Amyloid-beta overproduction causes abnormal mitochondrial dynamics via differential modulation of mitochondrial fission/fusion proteins. Proc. Natl. Acad. Sci. USA 105, 19318–19323. doi: 10.1073/pnas.0804871105 19050078 PMC2614759

[ref101] Westermann B. (2010). Mitochondrial fusion and fission in cell life and death. Nat. Rev. Mol. Cell Biol. 11, 872–884. doi: 10.1038/nrm3013, PMID: 21102612

[ref102] Yang Y. Chen J. H. Li L. Gao Y. S. Chen J. Fei Z. . (2013). Effect of different mild hypoxia manipulations on kainic acid-induced seizures in the hippocampus of rats. Neurochem. Res. 38, 123–132. doi: 10.1007/s11064-012-0899-6, PMID: 23065181

[ref103] Yang D. Y. Ying J. Wang X. F. Zhao T. C. Yoon S. T. Fang Y. . (2021). Mitochondrial dynamics: a key role in neurodegeneration and a potential target for neurodegenerative disease. Front. Neurosci. 15:654785. doi: 10.3389/fnins.2021.654785, PMID: 33912006 PMC8072049

[ref104] Yapa N. M. B. Lisnyak V. Reljic B. Ryan M. T. (2021). Mitochondrial dynamics in health and disease. FEBS Lett. 595, 1184–1204. doi: 10.1002/1873-3468.14077, PMID: 33742459

[ref105] Yin Z. Burger N. Kula-Alwar D. Aksentijević D. Bridges H. R. Prag H. A. . (2021). Structural basis for a complex I mutation that blocks pathological ROS production. Nat. Commun. 12:707. doi: 10.1038/s41467-021-20942-w 33514727 PMC7846746

[ref106] Zhan L. X. Wang T. Li W. Xu Z. C. Sun W. W. Xu E. (2010). Activation of Akt/FoxO signaling pathway contributes to induction of neuroprotection against transient global cerebral ischemia by hypoxic pre-conditioning in adult rats. J. Neurochem. 114, 897–908. doi: 10.1111/j.1471-4159.2010.06816.x, PMID: 20492357

[ref107] Zhang J. H. Qian H. Zhao P. Hong S. S. Xia Y. (2006). Rapid hypoxia preconditioning protects cortical neurons from glutamate toxicity through delta-opioid receptor. Stroke 37, 1094–1099. doi: 10.1161/01.STR.0000206444.29930.18, PMID: 16514101

[ref108] Zhang R. R. Xu M. X. Wang Y. Xie F. Zhang G. Qin X. Y. (2017). Nrf2-a promising therapeutic target for defensing against oxidative stress in stroke. Mol. Neurobiol. 54, 6006–6017. doi: 10.1007/s12035-016-0111-0, PMID: 27696223

[ref109] Zhen J. L. Wang W. P. Zhou J. J. Qu Z. Z. Fang H. B. Zhao R. R. . (2014). Chronic intermittent hypoxic preconditioning suppresses pilocarpine-induced seizures and associated hippocampal neurodegeneration. Brain Res. 1563, 122–130. doi: 10.1016/j.brainres.2014.03.032, PMID: 24680745

[ref110] Zorov D. B. Juhaszova M. Sollott S. J. (2014). Mitochondrial reactive oxygen species (Ros) and Ros-induced Ros release. Physiol. Rev. 94, 909–950. doi: 10.1152/physrev.00026.2013, PMID: 24987008 PMC4101632

