# Supplementary material for: Hypoxic preconditioning increases mitochondrial respiration and H2O2 production
Source: Front Mol Neurosci. 2025 Nov 19;18:1628567. doi: 10.3389/fnmol.2025.1628567 (PMC12673370; doi:10.3389/fnmol.2025.1628567)

Supplementary Materials - Figure S1

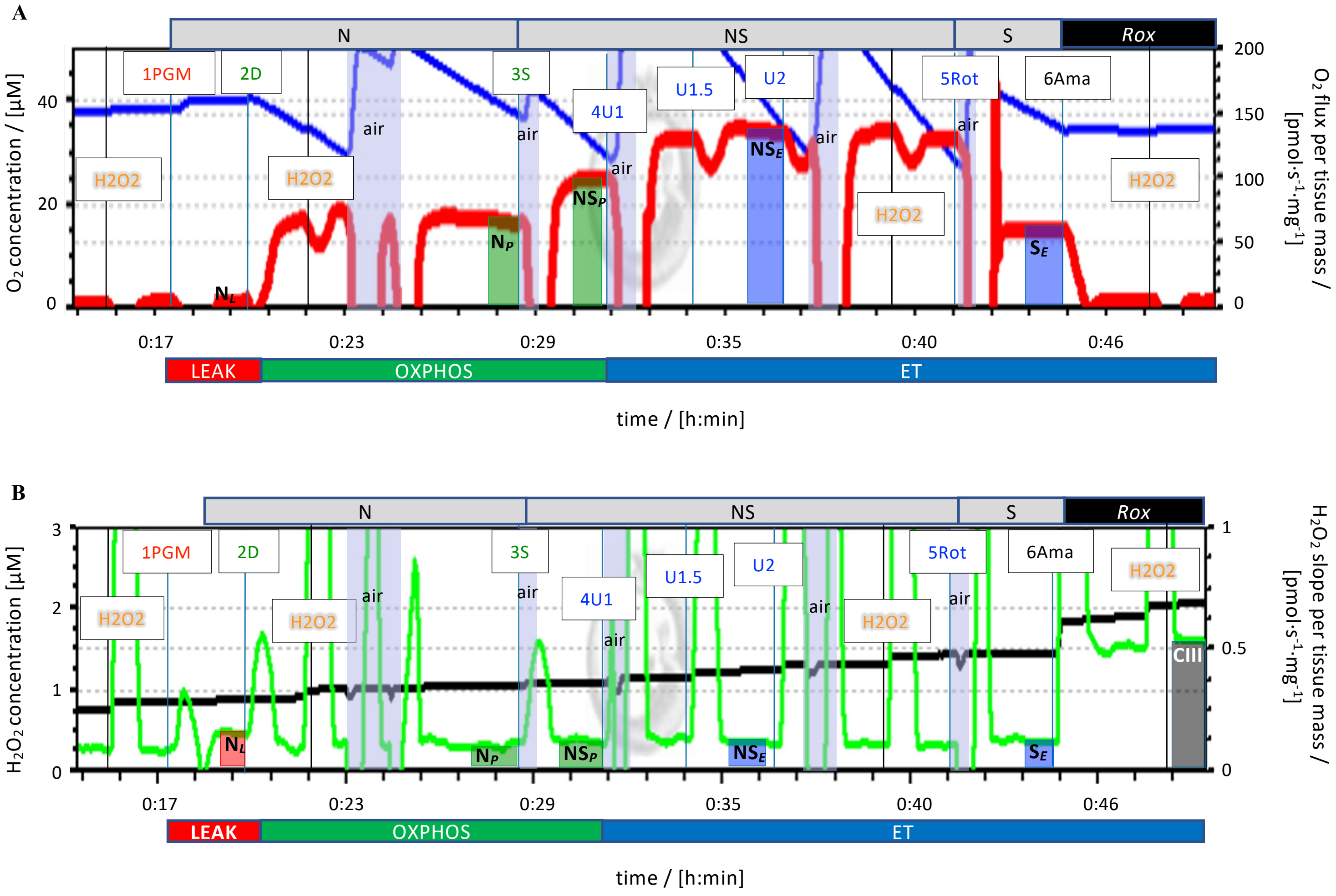

Supplementary Materials - Figure S2

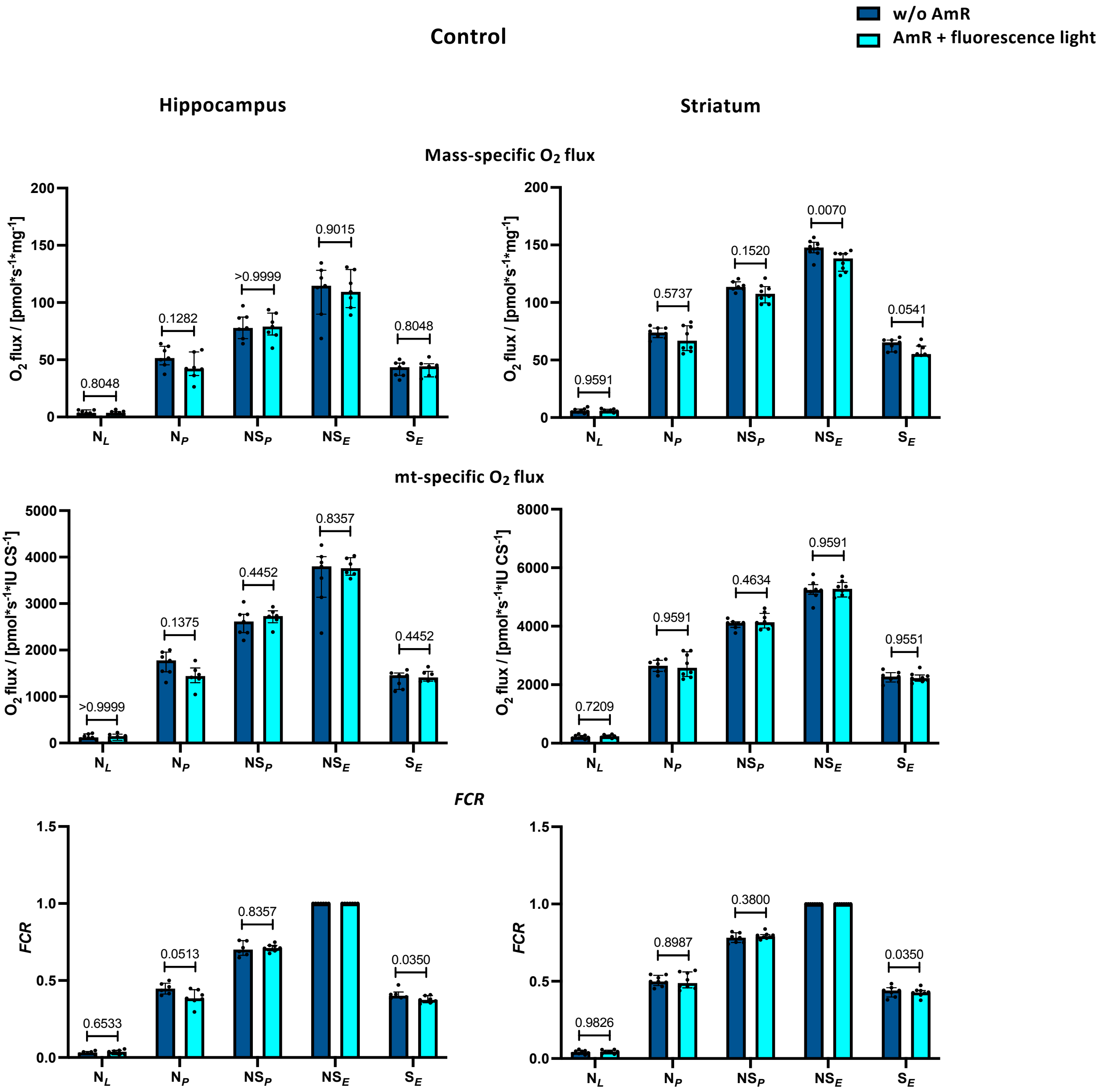

Supplementary Materials - Figure S3

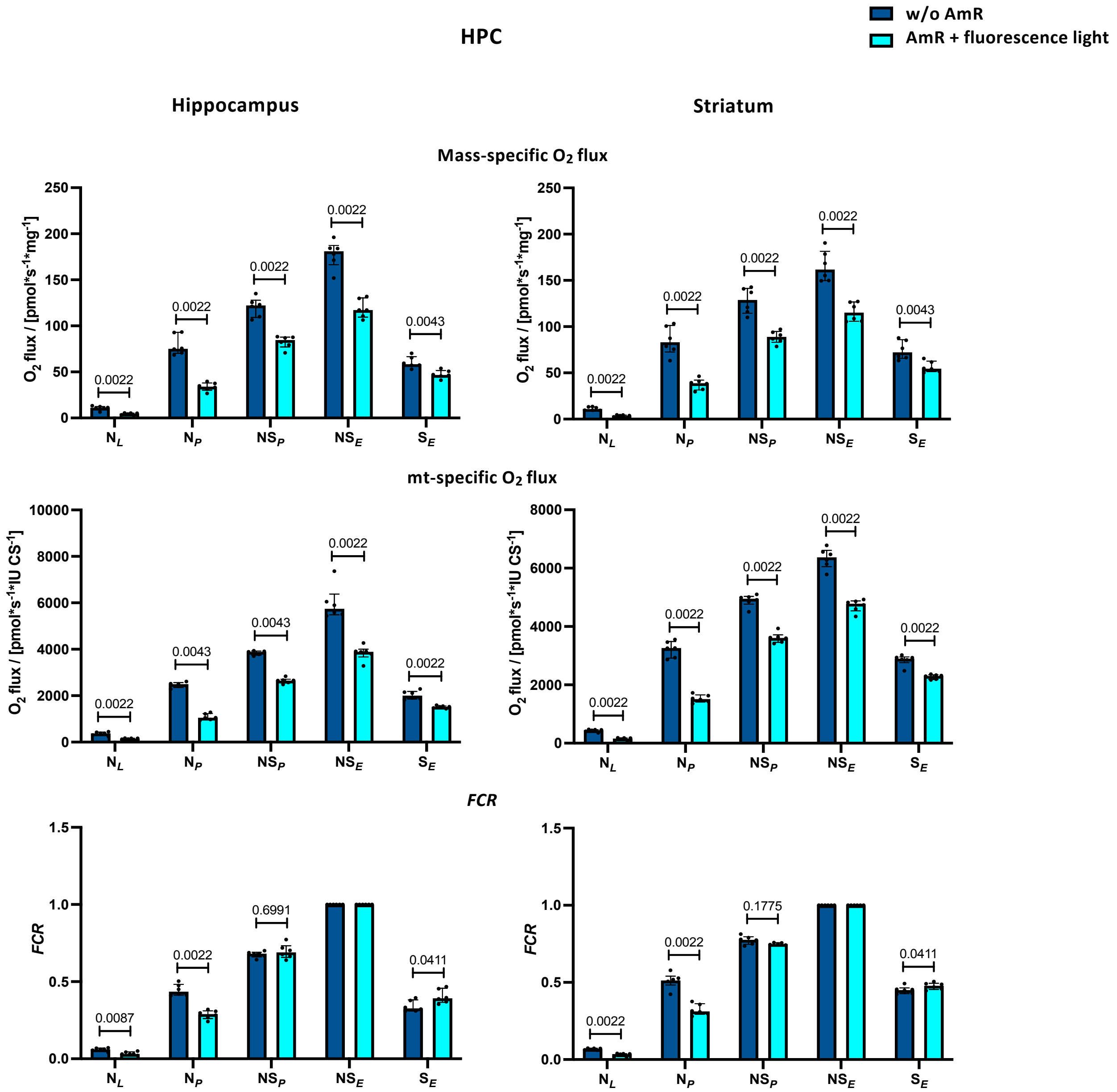

Supplementary Materials - Figure S4

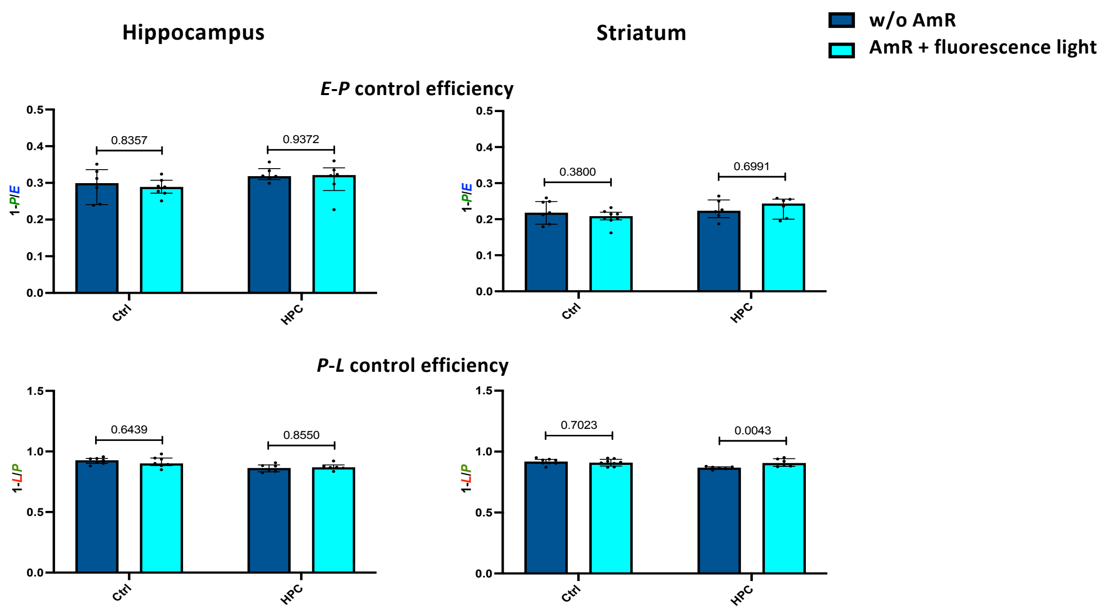

Supplementary Materials - Figure S5

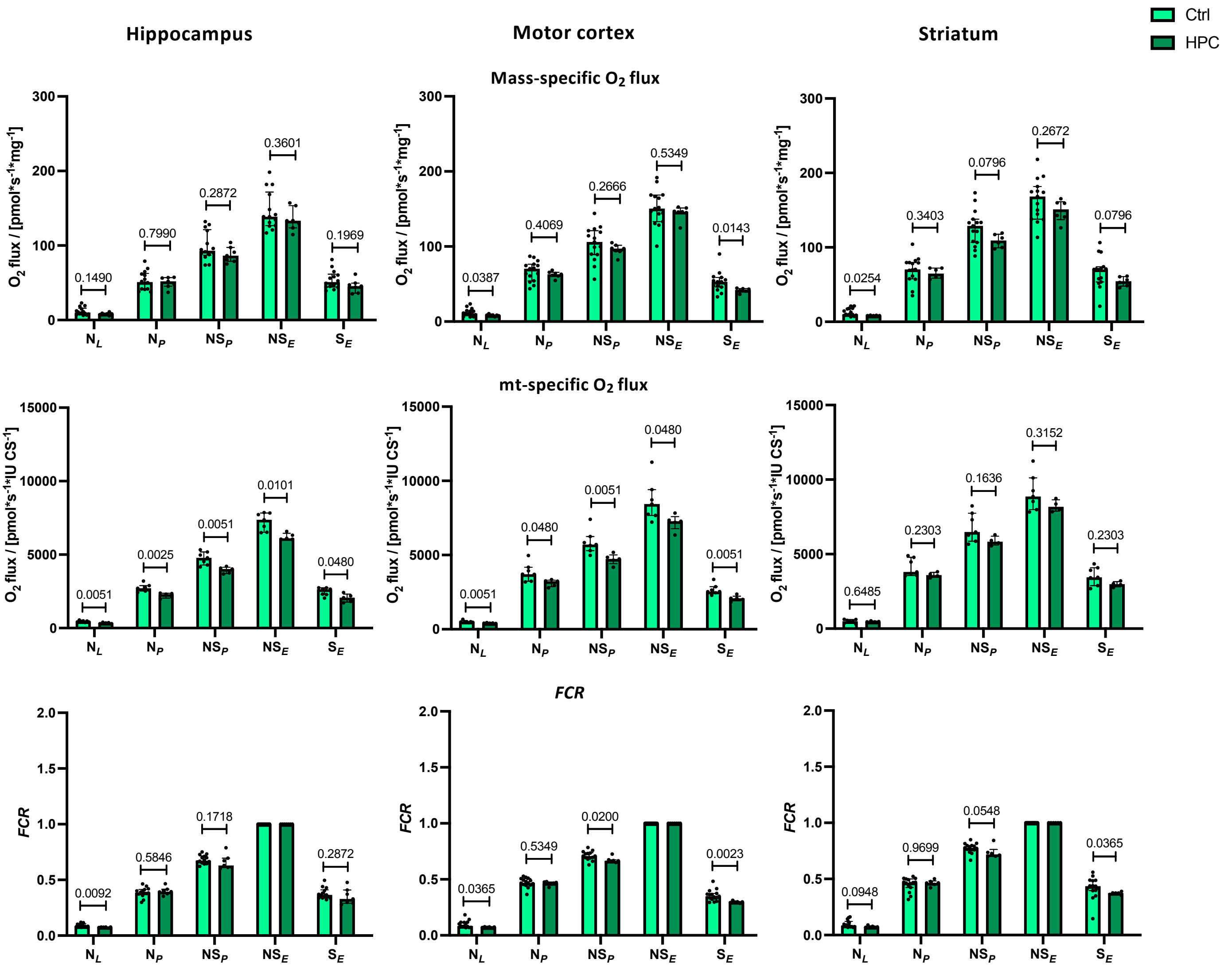

Supplementary Materials - Figure S6

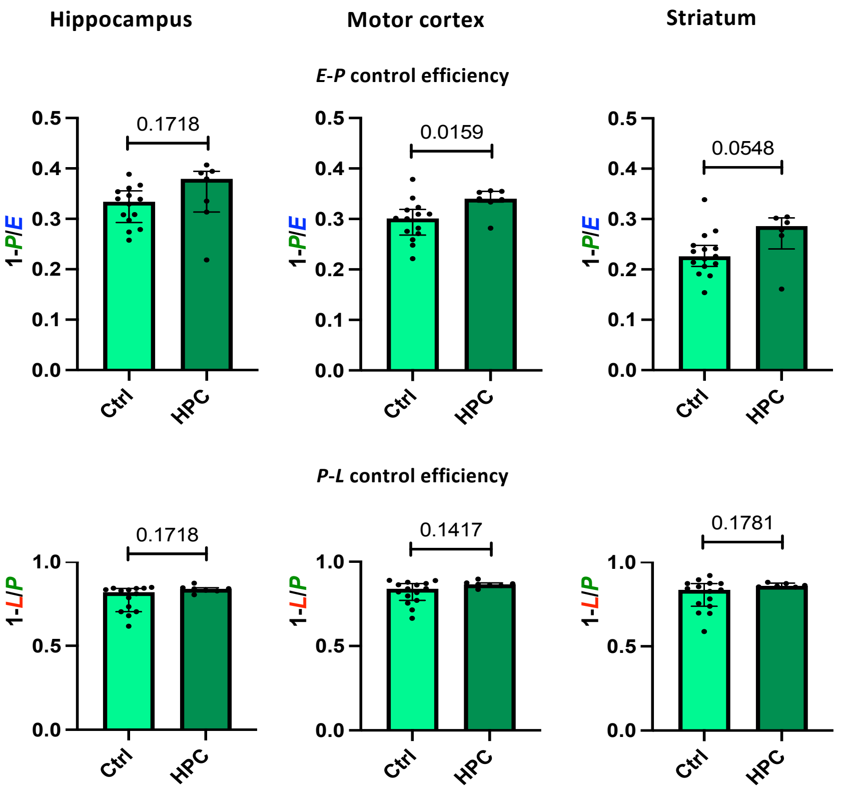

Supplementary Materials - Figure S7

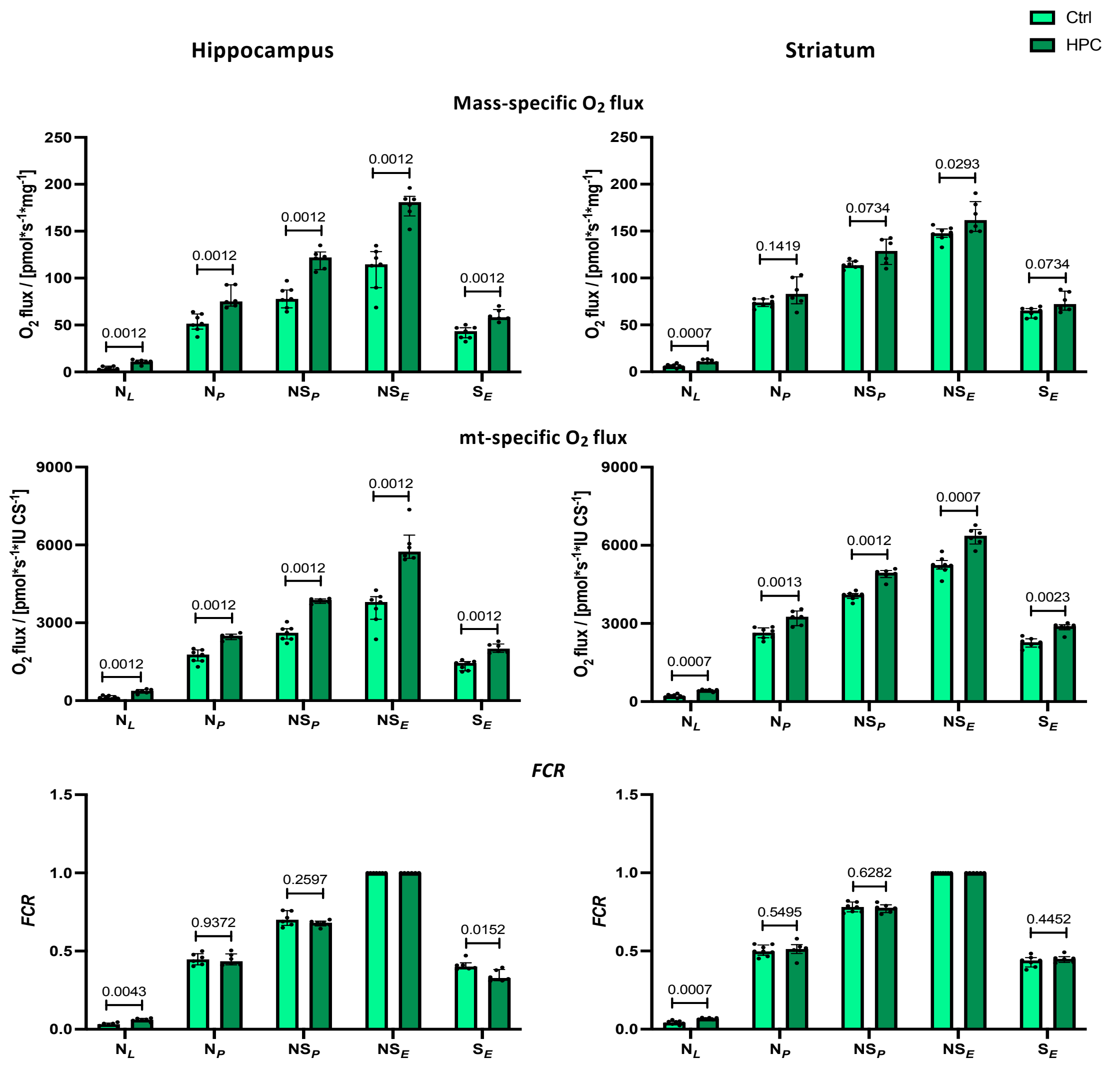

Supplementary Materials - Figure S8

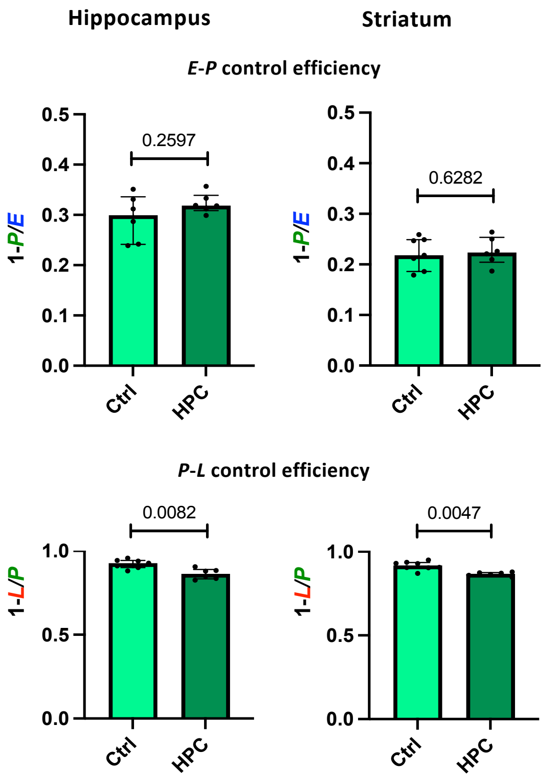

Supplementary Materials - Figure S9

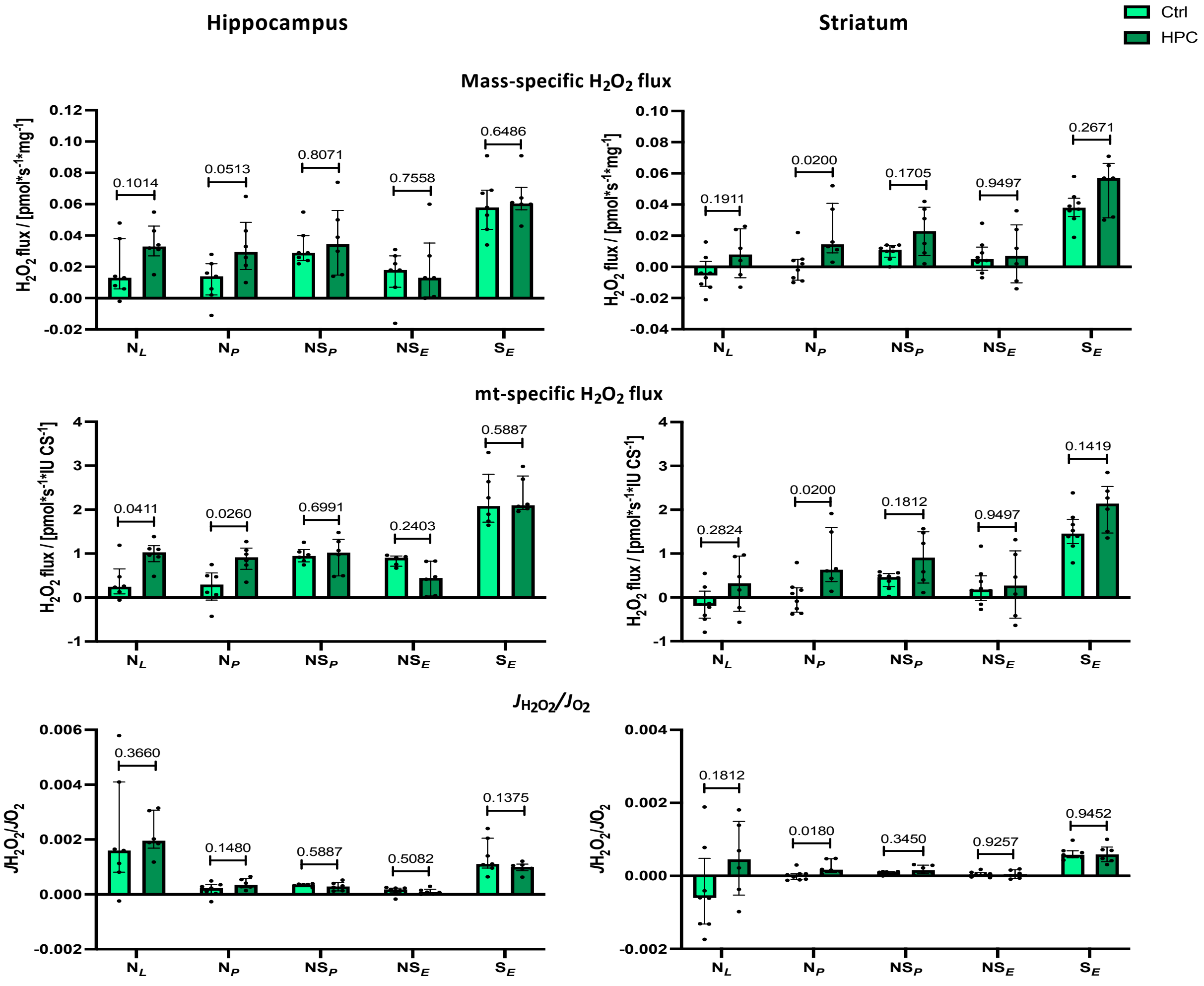

Supplementary Materials - Figure S10

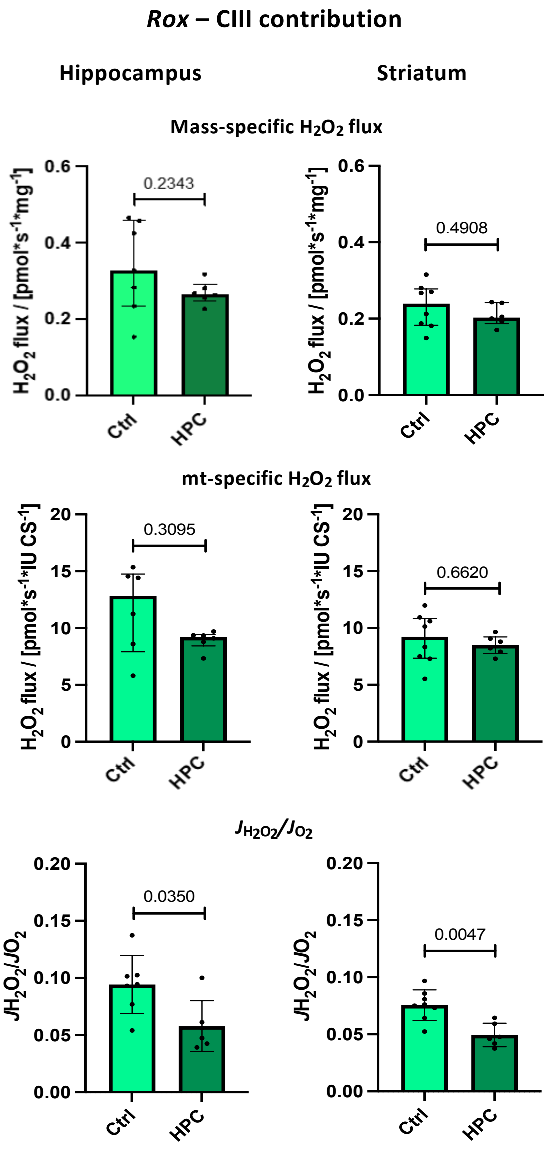

Supplementary Materials - Figure S11

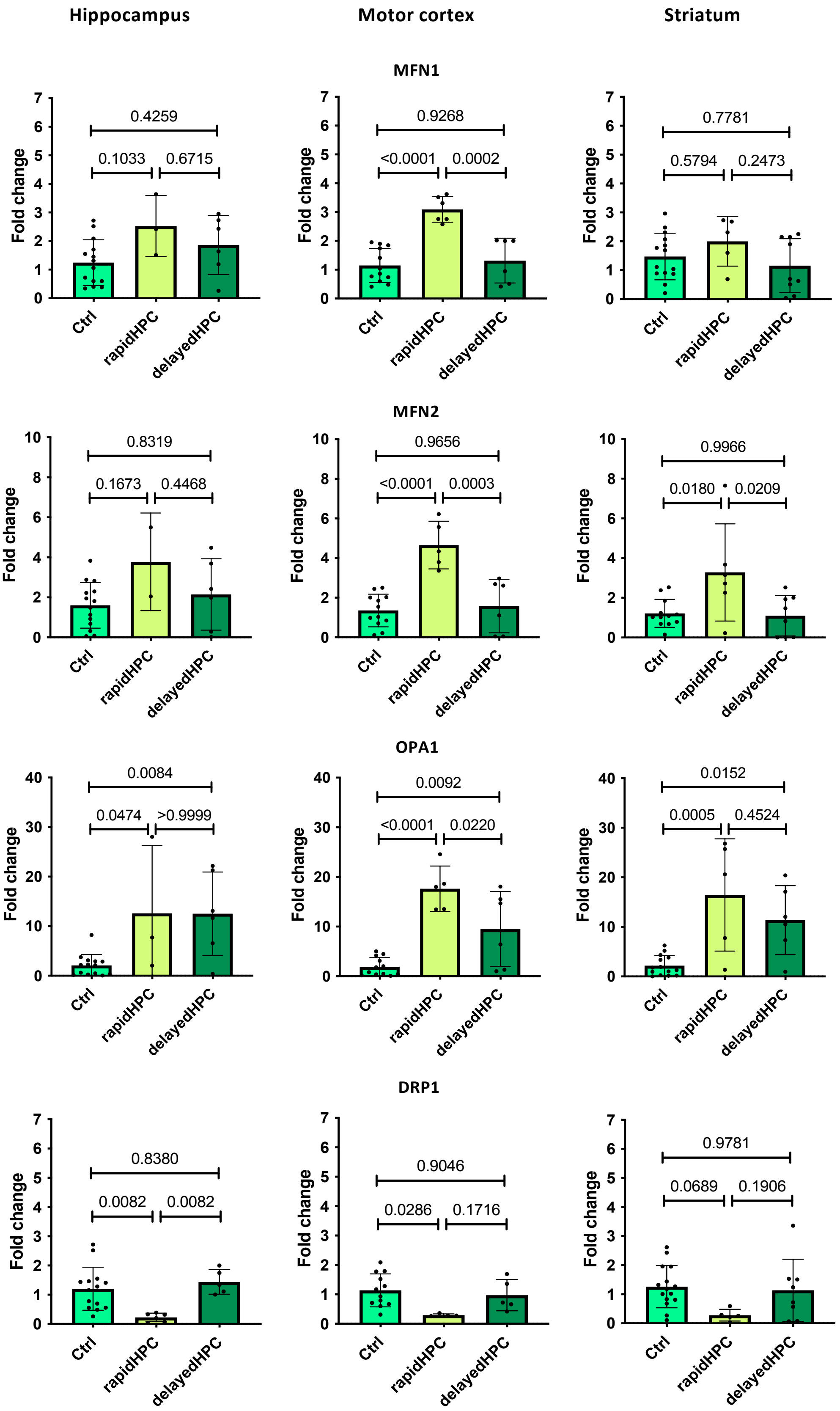

Supplement: SUPPLEMENTARY FIGURE S1 — Mitochondrial respiration and H2O2 production assessed under tissue normoxia (O2 concentration 30 to 40 μM). Representative traces of mitochondrial respiration (A) and H2O2 production (B) simultaneously measured by high-resolution respirometry (Oroboros FluoRespirometer). The experimental protocol closely matched the SUIT protocol in Figure 2 (main text). 1PMG: Sequential injections of pyruvate (P; 5 mM), malate (M; 2 mM), and glutamate (G; 10 mM) to initiate N-linked LEAK respiration NL. 2D: A kinetically saturating ADP concentration (D; 2.5 mM) induced N-linked OXPHOS NP. 3S: CII-substrate succinate (S; 50 mM) added to evaluate NS-pathway OXPHOS capacity NSP (CI- and CII-linked). 4U: Stepwise titrations of the uncoupler carbonyl cyanide chlorophenylhydrazone CCCP (U; 1.5–2.5 μM) to reach maximum respiration as an estimate of NS-pathway ET capacity NSE. 5Rot: Inhibition of CI by rotenone (Rot; 0.5 μM) to measure S-linked ET capacity SE. 6Ama: Inhibition of CIII by antimycin A (Ama; 2.5 μM) to assess residual oxygen consumption Rox for baseline correction of O2 flux in all mitochondrial respiratory states. During sections of reoxygenations, marked by shaded bars, fluxes cannot be measured. (A) The blue line shows O2 concentration [μM] with a zoom into the 50 μM range (compare Figure 2), the red line shows O2 flux per tissue wet mass [pmol·s−1·mg−1]. Marks (vertical bars) for evaluation of rates were set at O2 concentrations of 30 to 40 μM. O2 levels were adjusted by introducing N2 or H2 or opening the chambers. (B) Fluorometric signal (black line), calculated as equivalent H2O2 concentration [μM] (note that the actual H2O2 concentration is maintained at zero in the AmR assay). The green line denotes H2O2 flux per tissue mass [pmol·s−1·mg−1]. Changes of fluorescence sensitivity over time were assessed by sequentially titrating H2O2 (0.1 μM) in the course of the experiment. The black bar indicates the maximal contribution of CIII to ROS production. Titration spikes ar [file Data_Sheet_1.pdf]
